# Brentuximab vedotin plus chemotherapy for the treatment of front-line systemic anaplastic large cell lymphoma: subgroup analysis of the ECHELON-2 study at 5 years’ follow-up

**DOI:** 10.1038/s41408-025-01329-2

**Published:** 2025-08-01

**Authors:** Eva Domingo-Domènech, Barbara Pro, Tim Illidge, Steven Horwitz, Lorenz Trumper, Swami Iyer, Ranjana Advani, Nancy L. Bartlett, Jacob Haaber Christensen, Won-Seog Kim, Tatyana Feldman, Ilseung Choi, Giuseppe Gritti, David Belada, Andrei Shustov, Arpad Illes, Pier Luigi Zinzani, Andreas Hüttmann, Marek Trneny, Steven Le Gouill, Deepa Jagadeesh, Jonathan W. Friedberg, Meredith Little, Cassie Dong, Michelle Fanale, Keenan Fenton, Kerry J. Savage

**Affiliations:** 1 Department of Hematology, Institut Catala D’Oncologia, Hospital Duran i Reynals; Institut de reserca IDIBELL, L’Hospitalet de Llobregat, Barcelona, Spain; 2 Division of Hematology and Oncology, Department of Medicine, Northwestern University Feinberg School of Medicine, Chicago, IL USA; 3 NIHR Biomedical Research Centre, Manchester Academic Health Sciences Centre, Christie Hospital NHS Foundation Trust, University of Manchester, Manchester, UK; 4 Department of Medicine, Lymphoma Service, Memorial Sloan Kettering Cancer Center, New York, NY USA; 5 Universitätsmedizin Göttingen, Göttingen, Germany; 6 MD Anderson Cancer Center/University of Texas, Houston, TX USA; 7 Division of Oncology, Department of Medicine Division of Oncology, Stanford University, Stanford, CA USA; 8 Siteman Cancer Center, Washington University School of Medicine in St Louis, St. Louis, MO USA; 9 Odense University Hospital, Odense, Denmark; 10 Division of Hematology-Oncology, Department of Internal Medicine, Samsung Medical Center, Sungkyunkwan University School of Medicine, Seoul, Republic of Korea; 11 John Theurer Cancer Center, Hackensack Meridian Health, Hackensack, NJ USA; 12 Department of Hematology, National Hospital Organization Kyushu Cancer Center, Fukuoka, Japan; 13 Division of Hematology and Bone Marrow Transplant, ASST Papa Giovanni XXIII, Bergamo, Italy; 14 4th Department of Internal Medicine –Hematology, Charles University, Hospital and Faculty of Medicine, Hradec Králové, Czechia; 15 University of Washington Seattle Cancer Care Alliance & Fred Hutchinson Cancer Research Center, Seattle, WA USA; 16 Division of Hematology, Department of Internal Medicine, Faculty of Medicine, University of Debrecen, Debrecen, Hungary; 17 IRCCS Azienda Ospedaliero-Universitaria di Bologna, Istituto di Ematologia “Seràgnoli”, Bologna, Italy; 18 Dipartimento di Scienze Mediche e Chirurgiche, Università di Bologna, Bologna, Italy; 19 Universitatsklinikum Essen, Nordrhein-Westfalen, Germany; 20 First Faculty of Medicine at Charles University Hospital, Prague, Czechia; 21 Service d’hématologie, Institut Curie, Saint Cloud, France, Versailles, France; 22 Laboratoire d’Imagerie Translationnelle en Oncologie (LITO), U1288 Inserm/Institut Curie Centre de Recherche, Paris, France; 23 Université de Versailles Saint-Quentin (UVSQ), Versailles, France; 24 Department of Hematology and Medical Oncology, Taussig Cancer Institute, Cleveland Clinic, Cleveland, OH USA; 25 Wilmot Cancer Institute, University of Rochester, Rochester, NY USA; 26 Takeda Development Center Americas, Inc. (TDCA), Cambridge, MA USA; 27 Seagen Inc, Bothell, WA USA; 28 Centre for Lymphoid Cancer and Department of Medical Oncology, BC Cancer, Vancouver, BC Canada; 29 Present Address: Columbia University Irving Medical Center, New York, NY USA; 30 Present Address: Cellectar Biosciences Inc., New Jersey, NJ USA; 31 Present Address: Cogent Biosciences, Waltham, MA USA; 32 Present Address: Vertex Pharmaceuticals, Boston, MA USA

**Keywords:** T-cell lymphoma, Molecularly targeted therapy, Drug development

## Abstract

Trial registration: ClinicalTrials.gov number: NCT01777152

## Introduction

Peripheral T-cell lymphomas (PTCLs) represent ~10–15% of non-Hodgkin lymphomas [1, 2]. Systemic anaplastic large cell lymphoma (sALCL), a PTCL subtype characterized by universal CD30 expression [3, 4], is therefore a candidate for CD30-targeted treatment. sALCL is sub-divided by the presence or absence of anaplastic lymphoma kinase (ALK) protein [5]. ALK+ sALCL has a better prognosis than ALK– sALCL and other PTCL subtypes [5, 6]; nevertheless, 5-year overall survival (OS) remains 30–50% in older patients with ALK+ sALCL and those with other unfavorable prognostic factors [5, 7].

Brentuximab vedotin, an antibody–drug conjugate combining an anti-CD30 antibody with microtubule-disrupting agent monomethyl auristatin E, demonstrated high overall response (ORR) and complete remission (CR) rates of 86% and 57%, respectively, in a phase 2 study of patients with relapsed or refractory sALCL [8], leading to global approval [9]. The efficacy of front-line brentuximab vedotin in combination with cyclophosphamide, doxorubicin, and prednisone (A + CHP) in previously untreated patients with CD30-positive PTCL has been demonstrated in the phase 3 ECHELON-2 study, which enrolled 452 patients with CD30 + PTCL, of whom 316 (70%) had sALCL [7]. In all patients, progression was reduced by 30% with A + CHP versus cyclophosphamide, doxorubicin, vincristine, and prednisone (CHOP; hazard ratio [HR] 0.70, 95% confidence interval [CI] 0.53–0.91, *P* = 0.008); OS was also superior in patients treated with A + CHP (HR 0.72, 95% CI 0.53–0.99, *P* = 0.001) [10]. Given that ALK status is a significant predictor of outcomes in patients with sALCL, it is important to understand the longer-term impacts of ALK status on treatment outcomes to appropriately guide treatment decisions and management strategies [11]. Here, we present further analysis of ECHELON-2, detailing outcomes in ALK+ and ALK– sALCL subgroups at 5 years’ follow-up.

## Methods

### Study design and procedures

The phase 3, randomized, double-blind, placebo-controlled, multicenter ECHELON-2 study design has been published in full previously [7].

### Outcome measures

The primary efficacy outcome for this post-hoc analysis was progression-free survival (PFS; investigator-reported), defined as time from date of randomization to date of first documentation of relapse or progressive disease, death from any cause, or receipt of subsequent systemic chemotherapy to treat residual or progressive PTCL (per investigator). PFS was assessed in the overall sALCL cohort and in subgroups.

Other outcome measures included OS, ORR, CR rate at end of treatment (EOT) (according to Cheson 2007 [12]), and time to subsequent therapy (systemic therapy/palliative radiotherapy). Assessment of time to, duration of, and response to subsequent brentuximab vedotin treatment for recurrent disease were included as exploratory analyses.

PFS and OS were estimated using Kaplan–Meier methods. Differences between arms were analyzed by Cox proportional hazards regression modeling with nominal, descriptive *P*-values determined using a two-sided stratified log-rank test. Depending on the analysis, the log-rank test was stratified by ALK status (ALK + /ALK–) and International Prognostic Index (IPI) score (0/1 vs 2/3 vs 4/5) at randomization. Differences in ORR and CR rate between treatment arms were tested using the Cochran-Mantel-Haenszel test, stratified by the same factors.

### Ethics approval and consent to participate

The trial was performed in accordance with regulatory requirements and the ethical principles outlined in the Declaration of Helsinki. The protocol was approved by institutional review boards and independent ethics committees at individual sites (Supplementary Table 1). All patients provided written informed consent. Additional methods can be found in the Supplement.

## Results

### Patients

Overall, 316 patients with sALCL were randomized, including 98 (31%) ALK+ and 218 (69%) ALK– patients (Supplementary Fig. 1). The subgroup analysis of baseline demographic and clinical characteristics are provided in Supplementary Table 2.

### Efficacy

**Fig. 1:**
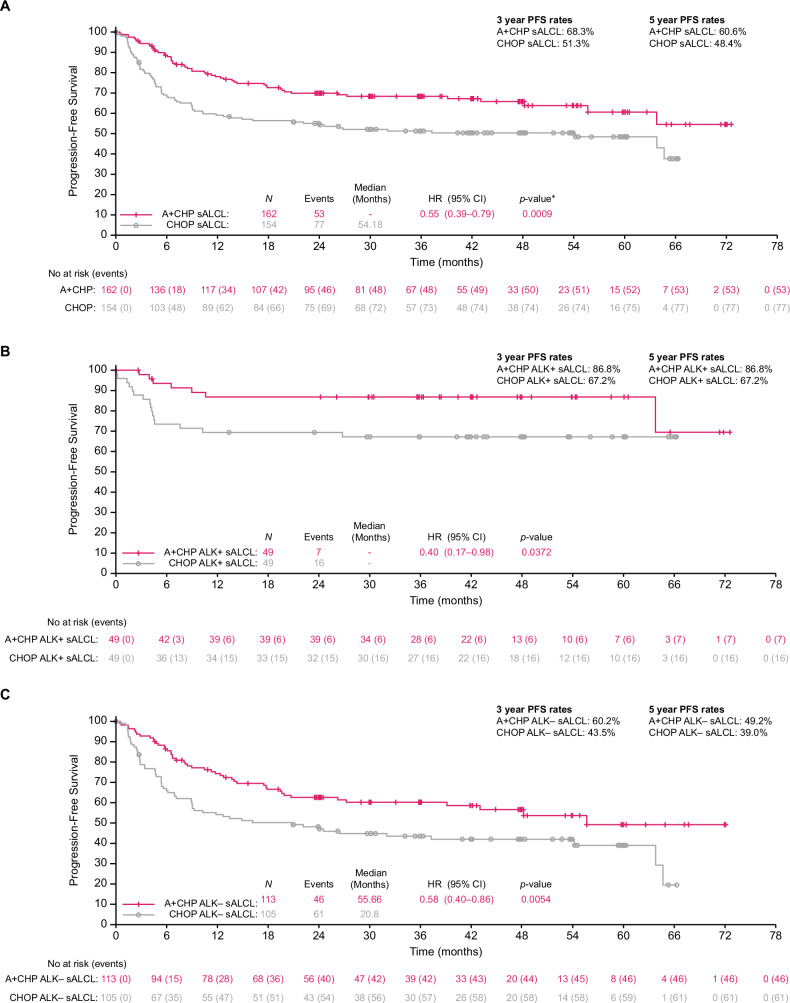
A+CHP provided a progression-free survival benefit over CHOP in patients with sALCL, including ALK+ and ALK– subgroups. Progression-free survival per investigator in patients with sALCL (**A**), and in the ALK+ (**B**) and ALK– (**C**) sALCL subgroups (intent-to-treat population, *N* = 316). *A* + *CHP* brentuximab vedotin plus cyclophosphamide, doxorubicin, and prednisone, ALK anaplastic lymphoma kinase, CHOP cyclophosphamide, doxorubicin, vincristine, and prednisone, CI confidence interval, HR hazard ratio, PFS progression-free survival, sALCL systemic anaplastic large cell lymphoma.

At data cut-off 2 October 2020, median (95% CI) duration of follow-up in patients with sALCL was 42.7 (41.9–47.8) months for PFS and 64.8 (61.9–67.1) months for OS. As previously reported, median PFS per investigator was not reached in the A + CHP arm and was 54.2 months in the CHOP arm, with estimated 5-year PFS rates of 61% versus 48%, respectively (HR 0.55, 95% CI 0.39–0.79; *P* = 0.0009 (Fig. 1A). A PFS benefit favoring A + CHP over CHOP was seen in both ALK+ (87% vs 67%; HR 0.40, 95% CI 0.17–0.98; *P* = 0.0372) and ALK– (49% vs 39%; HR 0.58, 95% CI 0.40–0.86; *P* = 0.0054) patients (Fig. 1B, C).

ORR at EOT was 90% in the A + CHP arm and 73% in the CHOP arm (difference: 17%, 95% CI 9–26%; *P* < 0.0001) (Table 1). The respective CR rates were 75% and 57% (difference: 18%, 95% CI 8–28%; *P* = 0.0006). In the A + CHP arm, the median time to first CR was 2.76 months (ALK + ) and 2.79 months (ALK–). The median time to first CR was 2.79 months for both patients that were ALK+ and ALK–, treated with CHOP. In ALK+ patients who achieved CR, the estimated 5-year PFS rate (95% CI) was 94.7% (80.6–98.7%) in the A + CHP arm and 93.3% (75.9–98.3%) in the CHOP arm. In patients with ALK– sALCL who achieved CR, the estimated 5-year PFS rate (95% CI) was 56.8% (40.5–70.2%) and 56.0% (39.3–69.7%) in the A + CHP and CHOP arms, respectively. The median duration of first CR was not reached for patients in ALK+ subgroup. In patients with ALK– sALCL, median duration of first CR was 52.7 months and 48.6 months in the A + CHP and CHOP arms, respectively. ORR and CR rates for the ALK+ and ALK– subgroups were higher with A + CHP versus CHOP (ALK + : 94% and 82% vs 76% and 61%; ALK–: 88% and 73% vs 71% and 55%; Table 1). Progressive disease was less common in the A + CHP arm, occurring in 4% of patients (2% of ALK+ and 4% of ALK– patients) and 14% of those in the CHOP arm (18% of ALK+ and 12% of ALK– patients). The median duration of objective response was 52.7 months for A + CHP and 51.4 months for CHOP.

**Table 1:** Best response per Cheson 2007 [12] at the end of treatment in patients with sALCL (intent-to-treat population, *N* = 316).

	A + CHP			CHOP		
Objective response, *n* (%) [95% CI]	ALK + sALC *n* = 49	ALK– sALCL *n* = *113*	Overall *n* = 162	ALK + sALCL *n* = 49	ALK– sALCL *n* = *105*	Overall *n* = 154
Overall response rate	46 (94) [83–99]	100 (88) [81–94]	146 (90) [85–94]	37 (76) [61–87]	75 (71) [62–80]	112 (73) [65–80]
Complete remission	40 (82) [68–91]	82 (73) [63–81]	122 (75) [68–82]	30 (61) [46–75]	58 (55) [45–65]	88 (57) [49–65]
Partial remission	6 (12)	18 (16)	24 (15)	7 (14)	17 (16)	24 (16)
Stable disease	0	3 (3)	3 (2)	0	3 (3)	3 (2)
Progressive disease	1 (2)	5 (4)	6 (4)	9 (18)	13 (12)	22 (14)
Not evaluable	2 (4)	5 (4)	7 (4)	3 (6)	14 (13)	17 (11)

*A* + *CHP* brentuximab vedotin plus cyclophosphamide, doxorubicin, and prednisone, *ALK* anaplastic lymphoma kinase, *CHOP* cyclophosphamide, doxorubicin, vincristine, and prednisone, *CI* confidence interval, *sALCL* systemic anaplastic large cell lymphoma.

As previously reported, after 5-years’ follow-up, median OS was not reached in either treatment arm, but OS favored A + CHP versus CHOP (HR 0.66, 95% CI 0.43–1.01; *P* = 0.053; Supplementary Fig. 4A) with 5-year OS rates of 76% versus 69% [10] in all sALCL. OS analysis in ALK+ and ALK– was limited by infrequent events, especially in ALK+ sALCL (Supplementary Fig. 4B, C); this OS benefit was also demonstrated in the subgroup analyses (Supplementary Fig. 5B, C).

Overall, 35 of 162 patients (22%) in the A + CHP arm and 58 of 154 patients (38%) in the CHOP arm received subsequent new anticancer therapy, with the majority (94% A + CHP; 100% CHOP) receiving systemic therapies for residual or progressive disease consistent with a PFS event. In the A + CHP and CHOP arms, 18 (11%) and 39 (25%) patients, respectively, received brentuximab vedotin as a subsequent therapy (Supplementary Table 4).

### Safety

Incidence and severity of any-grade and grade ≥3 treatment-emergent adverse events (TEAEs) were generally comparable between arms (Supplementary Table 5). The most common any-grade TEAEs (≥20% incidence in either arm) were peripheral sensory neuropathy (48% and 42% in A + CHP and CHOP arms, respectively), nausea (46% and 42%, respectively), neutropenia (37% and 38%), diarrhea (34% and 23%), vomiting (28% and 19%), constipation (26% and 29%), and fatigue (23% and 19%). TEAEs led to treatment discontinuation in 6 patients (4%) in the A + CHP arm and 14 (9%) in the CHOP arm (Supplementary Table 5). Additional results can be found in the Supplement.

## Discussion

Consistent with the 5-year study in sALCL [10], improved outcomes were observed with A + CHP versus CHOP in the ALK+ and ALK–subgroups. The risk of PFS events was reduced by 45% in the overall sALCL cohort, with a 42% reduction in ALK– patients (*n* = 218) and a 60% reduction in ALK+ patients (*n* = 98). In both arms, 5-year PFS rates were higher in ALK+ versus ALK– patients (87% vs 49% A + CHP; 67% vs 39% CHOP). The observed greater treatment benefit in ALK+ versus ALK– patients is unsurprising (even excluding ALK+ patients with IPI score <2). ALK+ sALCL is known to be associated with improved prognosis, partly due to the younger age of presentation [5]; previous studies showed improved survival with CHOP-based chemotherapy [6, 13]. This analysis demonstrates that the superior efficacy of A + CHP in patients with sALCL is driven by improvements in both ALK+ and ALK– patients.

Cases of ALK − ALCL with a *DUSP22* rearrangement (*DUSP22r*) have been recognized by the International Consensus Classification as a genetic entity based on distinct pathological and molecular features [14]. *DUSP22r* ALK − ALCL is usually associated with a favorable prognosis, with 5-year OS rates ranging from 80 to 90% [15–18], although some studies have demonstrated poorer prognoses [15, 19, 20], with clinical factors also playing a role [17]. This is an important adjunct test alongside *P63* rearrangement, which is associated with a poor prognosis. Unfortunately, this information was not available for the ECHELON-2 study.

Excluding ALK + ALCL, a *post hoc* analysis showed improved PFS with consolidative autologous stem cell transplant in patients who achieved a CR after CHP-brentuximab vedotin, including in the subset of ALK − ALCL patients [21]. Although randomized trials are ongoing (NCT05444712 [22]) and low-risk cases such as those with *DUSP2*2*r* may be considered for chemotherapy alone, consolidative transplant should still be a treatment consideration in most patients with ALK − ALCL.

The safety profile of A + CHP in patients with sALCL was consistent with that in the overall ECHELON-2 population [10], with no evidence of increased toxicity versus CHOP. Neutropenia rates were similar between treatment arms and use of granulocyte-colony stimulating factor (G-CSF) prophylaxis reduced the risk of febrile neutropenia, as previously reported [23]. Further, peripheral neuropathy rates were similar between treatment arms, and most cases had resolved at 5-years’ follow up.

Older patients (aged ≥60 years) receiving A + CHP had comparable safety outcomes to those receiving CHOP, with similar rates of any grade and grade ≥3 TEAEs. Although there was a numerically higher risk of febrile neutropenia in older patients treated with A + CHP, this was substantially reduced with G-CSF primary prophylaxis, which is now recommended for all patients, regardless of age. The OS and PFS rates were only slightly lower in older patients compared to those aged <60 years old and taken together with the safety findings and updated management strategies, this indicates that A + CHP may be suitable for eligible patients, regardless of age. As previously reported, patients retreated with brentuximab vedotin achieved meaningful response rates which were not impacted by time from previous A + CHP therapy [10]. This suggests that brentuximab vedotin may be considered a treatment option following A + CHP and may be as a useful bridge to SCT in some patients.

In conclusion, this analysis of the long-term efficacy and safety of the A + CHP regimen further supports its use for the front-line treatment of patients with sALCL, in older and younger, and ALK+ and ALK- patients. Future research should explore additional biomarkers and patient characteristics to further optimize treatment pathways. Prospective analyses of this nature could inform a more personalized therapeutic approached for patients with A + CHP.

## Supplementary information

Supplementary material

## Data Availability

Upon request and subject to review, Pfizer will provide the data that support the findings of this study. Subject to certain criteria, conditions, and exceptions, Pfizer may also provide access to the related individual de-identified participant data. See https://www.pfizer.com/science/clinical-trials/trial-data-and-results for more information.
