# Correction: Brentuximab vedotin plus chemotherapy for the treatment of front-line systemic anaplastic large cell lymphoma: subgroup analysis of the ECHELON-2 study at 5 years’ follow-up

**DOI:** 10.1038/s41408-025-01432-4

**Published:** 2026-01-28

**Authors:** Eva Domingo-Domènech, Barbara Pro, Tim Illidge, Steven Horwitz, Lorenz Trumper, Swami Iyer, Ranjana Advani, Nancy L. Bartlett, Jacob Haaber Christensen, Won-Seog Kim, Tatyana Feldman, Ilseung Choi, Giuseppe Gritti, David Belada, Andrei Shustov, Arpad Illes, Pier Luigi Zinzani, Andreas Hüttmann, Marek Trneny, Steven Le Gouill, Deepa Jagadeesh, Jonathan W. Friedberg, Meredith Little, Cassie Dong, Michelle Fanale, Keenan Fenton, Kerry J. Savage

**Affiliations:** 1 Department of Hematology, Institut Catala D’Oncologia, Hospital Duran i Reynals; Institut de reserca IDIBELL, L’Hospitalet de Llobregat, Barcelona, Spain; 2 Division of Hematology and Oncology, Department of Medicine, Northwestern University Feinberg School of Medicine, Chicago, IL USA; 3 NIHR Biomedical Research Centre, Manchester Academic Health Sciences Centre, Christie Hospital NHS Foundation Trust, University of Manchester, Manchester, UK; 4 Department of Medicine, Lymphoma Service, Memorial Sloan Kettering Cancer Center, New York, NY USA; 5 Universitätsmedizin Göttingen, Göttingen, Germany; 6 MD Anderson Cancer Center/University of Texas, Houston, TX USA; 7 Division of Oncology, Department of Medicine Division of Oncology, Stanford University, Stanford, CA USA; 8 Siteman Cancer Center, Washington University School of Medicine in St Louis, St. Louis, MO USA; 9 Odense University Hospital, Odense, Denmark; 10 Division of Hematology-Oncology, Department of Internal Medicine, Samsung Medical Center, Sungkyunkwan University School of Medicine, Seoul, Republic of Korea; 11 John Theurer Cancer Center, Hackensack Meridian Health, Hackensack, NJ USA; 12 Department of Hematology, National Hospital Organization Kyushu Cancer Center, Fukuoka, Japan; 13 Division of Hematology and Bone Marrow Transplant, ASST Papa Giovanni XXIII, Bergamo, Italy; 14 4th Department of Internal Medicine –Hematology, Charles University, Hospital and Faculty of Medicine, Hradec Králové, Czechia; 15 University of Washington Seattle Cancer Care Alliance & Fred Hutchinson Cancer Research Center, Seattle, WA USA; 16 Division of Hematology, Department of Internal Medicine, Faculty of Medicine, University of Debrecen, Debrecen, Hungary; 17 IRCCS Azienda Ospedaliero-Universitaria di Bologna, Istituto di Ematologia “Seràgnoli”, Bologna, Italy; 18 Dipartimento di Scienze Mediche e Chirurgiche, Università di Bologna, Bologna, Italy; 19 Universitatsklinikum Essen, Nordrhein-Westfalen, Germany; 20 First Faculty of Medicine at Charles University Hospital, Prague, Czechia; 21 Service d’hématologie, Institut Curie, Saint Cloud, France, Versailles, France; 22 Laboratoire d’Imagerie Translationnelle en Oncologie (LITO), U1288 Inserm/Institut Curie Centre de Recherche, Paris, France; 23 Université de Versailles Saint-Quentin (UVSQ), Versailles, France; 24 Department of Hematology and Medical Oncology, Taussig Cancer Institute, Cleveland Clinic, Cleveland, OH USA; 25 Wilmot Cancer Institute, University of Rochester, Rochester, NY USA; 26 Takeda Development Center Americas, Inc. (TDCA), Cambridge, MA USA; 27 Seagen Inc, Bothell, WA USA; 28 Centre for Lymphoid Cancer and Department of Medical Oncology, BC Cancer, Vancouver, BC Canada; 29 Present Address: Columbia University Irving Medical Center, New York, NY USA; 30 Present Address: Cellectar Biosciences Inc., New Jersey, NJ USA; 31 Present Address: Cogent Biosciences, Waltham, MA USA; 32 Present Address: Vertex Pharmaceuticals, Boston, MA USA

**Keywords:** T-cell lymphoma, Molecularly targeted therapy, Drug development

Correction to: *Blood Cancer Journal* 10.1038/s41408-025-01329-2, published online 01 August 2025

Following the publication of this article, the authors noted the following sentence could be misinterpreted: ‘In patients with ALK– sALCL, the estimated 5-year PFS rate (95% CI) was 56.8% (40.5–70.2%) and 56.0% (39.3–69.7%) in the A + CHP and CHOP arms, respectively’. This sentence is in fact about patients with ALK– sALCL who achieved a complete response (CR), and while the sentence appears in the context of a paragraph discussing patients with a CR, the following correction has been made to avoid confusion: ‘In patients with ALK– sALCL who achieved CR, the estimated 5-year PFS rate (95% CI) was 56.8% (40.5–70.2%) and 56.0% (39.3–69.7%) in the A + CHP and CHOP arms, respectively’. Data showing 5-year PFS in patients with ALK– disease, regardless of response, appear in Figure 1.

The original article has been corrected.

