# Supplementary material for: Brentuximab vedotin plus chemotherapy for the treatment of front-line systemic anaplastic large cell lymphoma: subgroup analysis of the ECHELON-2 study at 5 years’ follow-up
Source: Blood Cancer J. 2025 Aug 1;15(1):129. doi: 10.1038/s41408-025-01329-2 (PMC12317119; doi:10.1038/s41408-025-01329-2)
Supplement: Supplementary file 1 — Supplementary material [file 41408_2025_1329_MOESM1_ESM.docx]

**SUPPLEMENTARY MATERIAL**

**Brentuximab vedotin plus chemotherapy for the treatment of front-line systemic anaplastic large cell lymphoma: subgroup analysis of the ECHELON-2 study at 5 years’ follow-up**

**Authors:** Eva Domingo-Domènech^1^, Barbara Pro^2*,^ Tim Illidge^3^, Steven Horwitz^4^, Lorenz Trumper^5^, Swami Iyer^6^, Ranjana Advani^7^, Nancy L. Bartlett^8^, Jacob Haaber Christensen^9^, Won-Seog Kim^10^, Tatyana Feldman^11^, Ilseung Choi^12^, Giuseppe Gritti^13^, David Belada^14^, Andrei Shustov^15**^, Arpad Illes^16^, Pier Luigi Zinzani^17,18^, Andreas Hüttmann^19^, Marek Trneny^20^, Steven Le Gouill^21^, Deepa Jagadeesh^22^, Jonathan W. Friedberg^23^, Meredith Little^24^,*** Cassie Dong^24^,**** Michelle Fanale^25^, Keenan Fenton^25^ and Kerry J. Savage^26^

**SUPPLEMENTARY METHODS**

Eligible patients were aged ≥18 years with previously untreated peripheral T-cell lymphoma (according to World Health Organization 2008 classification criteria [1]), with CD30 expression on ≥10% of cells (per local review) and Eastern Cooperative Oncology Group performance status (ECOG PS) ≤2. Target enrolment was 450 patients, including 75 ± 5% with systemic anaplastic large cell lymphoma (sALCL). Patients with anaplastic lymphoma kinase (ALK)+ status (per local pathology review) were eligible if they had an International Prognostic Index (IPI) score ≥2. Patients were randomized 1:1 to receive brentuximab vedotin in combination with cyclophosphamide, doxorubicin, and prednisone (A+CHP) or cyclophosphamide, doxorubicin, vincristine, and prednisone (CHOP) every 3 weeks for 6–8 cycles. The use of granulocyte-colony stimulating factor (G-CSF) and/or chemotherapy for stem-cell collection for a future autologous stem cell transplant was permitted per institution standard after end of treatment (EOT) procedures were completed. Post-treatment consolidative stem cell transplantation (SCT) or radiotherapy were permitted at the investigator’s discretion. Use of G-CSF was also permitted as primary prophylaxis for febrile neutropenia per investigator discretion.

Safety was assessed based on rates of any treatment-emergent adverse events (TEAEs), treatment-related TEAEs, grade ≥3 TEAEs, serious TEAEs, TEAEs leading to discontinuation, and deaths. TEAEs were summarized for the overall sALCL population and by age subgroup (<60 vs ≥60 years) and ALK status (ALK+ vs ALK–). Incidence/severity of peripheral neuropathy (PN) was recorded, and resolution of cases during follow-up was assessed. TEAEs of interest included febrile neutropenia, neutropenia, and grade ≥3 infections, which were assessed in the overall sALCL population, in patients aged <60 and ≥60 years, and in patients with/without G-CSF primary prophylaxis.

**SUPPLEMENTARY RESULTS**

**Efficacy**

Forty-five patients with sALCL in the A+CHP arm and 57 in the CHOP arm had progressed following treatment. Median overall survival (OS) from randomization among these patients was 34 months in the A+CHP arm and 36 months in CHOP arm, with estimated 5-year OS rates (95% confidence interval [CI]) of 39% (24.7–53.1) and 46% (32.3–57.8), respectively. Median OS was 70.4 months in patients ≤60 years old and was not reached in patients >60 years old. Estimated 5-year OS rates were higher in patients <60 years old, at 85.4% (A+CHP arm) and 78.5% (CHOP arm) (hazard ratio [HR]: 0.60, 95% CI: 0.31–1.18) compared to patients ≥60 years, at 59.7% (A+CHP arm) and 49.8% (CHOP arm) (HR: 0.71, 95% CI: 0.41–1.22) (Supplementary Figure 3A).

Subgroup analyses of progression-free survival (PFS) by age, disease indication subtype (ALK+ and ALK–), and IPI score also generally favored A+CHP over CHOP (HRs <1) (Supplementary Figure 2). Median PFS was 25.4 months in patients ≤60 years old and was not reached in patients >60 years old. Estimated 5-year PFS rates were higher in patients <60 years old, at 75.5% (A+CHP arm) and 55.9% (CHOP arm) (HR: 0.42, 95% CI: 0.25–0.70) compared to patients ≥60 years, at 40.2% (A+CHP arm) and 34.7% (CHOP arm) (HR: 0.76, 95% CI: 0.47–1.25) (Supplementary Figure 2A). The impact of IPI score was explored in ALK+ and ALK– subgroups (Supplementary Figure 3; Supplementary Figure 2B–C). In patients with ALK+ sALCL (restricted to those with IPI score ≥2 by eligibility criteria; A+CHP, *n* = 49; CHOP, *n* = 49), the HR for PFS with A+CHP versus CHOP was 0.32 (95% CI 0.12–0.90) in patients with IPI score 2–3 (*n =* 43 in each arm) and 0.99 (95% CI 0.14–7.06) in those with IPI score 4–5 (A+CHP, *n =* 5; CHOP, *n =* 6); notably, patient numbers in the high-risk group were small (Supplementary Figure 2; Supplementary Figure 3A–B). In the ALK– subgroup (A+CHP, *n =* 113; CHOP, *n =* 105), a PFS benefit favoring A+CHP was observed in the IPI 0–1 group (HR 0.39, 95% CI 0.17–0.89) with a trend observed in the higher risk groups (IPI 2–3: HR 0.65, 95% CI 0.38–1.11; IPI 4–5: HR 0.69, 95% CI 0.32–1.51) and age subgroups (Supplementary Figure 2; Supplementary Figure 3C–E;). In ALK– patients analyzed by sex, the PFS HR (95% CI) with A+CHP versus CHOP in female patients was 0.30 (0.14–0.61; A+CHP, *n* = 49; CHOP, *n* = 25) compared with 0.81 (0.52–1.28; A+CHP, *n* = 64 CHOP, *n* = 80) in male patients (Supplementary Figure 2C).

In the A+CHP and CHOP arms, 29 of 162 patients (18%) and 22 of 154 patients (14%), respectively, had relapsed/recurrent disease (defined as disease relapse after achieving a complete response [CR] at EOT), of whom 21 patients in each arm received subsequent anti-cancer therapy (Supplementary Table 3). When considering all patients who received subsequent treatment, time to subsequent therapy was longer in the A+CHP arm compared with the CHOP arm (HR 0.45, 95% CI 0.30–0.69; *P =* 0.0002; Supplementary Figure 6). Median time to subsequent therapy was not reached in either group and one patient (5%) in the CHOP arm received additional treatment with brentuximab vedotin before disease progression. After first relapse/recurrence of disease, 15 (52%) patients in the A+CHP arm and 14 (64%) in the CHOP arm were still alive.

As previously reported [2], 58 sALCL patients (19 in the A+CHP arm [18 ALK– and 1 ALK+ patients] and 39 in the CHOP arm [32 ALK– and 7 ALK+ patients]) received subsequent brentuximab vedotin, including 51 (17 and 34, respectively) receiving it as monotherapy and 7 (2 and 5, respectively) within a combination regimen. Median time from the start of randomized treatment to subsequent treatment with brentuximab vedotin was 15.0 months in the A+CHP arm and 7.7 months in the CHOP arm. Subsequent brentuximab vedotin was administered for a median duration of 2.3 months in each arm. For patients receiving brentuximab vedotin as the first subsequent treatment after initial A+CHP or CHOP, by investigator account, the overall response rate (ORR) was 60% and CR rate was 34%; in ALK– patients, ORR was 56% and CR rate was 32%, whereas in ALK+ patients, ORR was 88% and CR rate was 50%. Similar results were noted in patients receiving brentuximab vedotin as the first subsequent treatment up to 12 months (*n* = 45) versus more than 12 months (*n* = 13) after end of initial therapy with brentuximab vedotin (ORR 60% [*n* = 27] vs 63% [*n* = 8]; CR rates 36% [*n* = 16] vs 31% [*n* = 4]).

Among the 58 patients who received brentuximab vedotin as the first subsequent treatment, 15 patients went on to receive a second treatment with brentuximab vedotin treatment; ORR was 40% (*n* = 6); all six patients had a CR.

In the A+CHP arm, 5 patients received consolidative SCT after frontline therapy; 3 patients received consolidative SCT after frontline therapy in the CHOP arm.

**Safety**

The safety population comprised 314 patients with sALCL who received randomized study treatment (excluding 2 patients randomized to A+CHP due to death before first dose and not being eligible for randomization). Patients received a median (range) of 6 (1–8) treatment cycles for both A+CHP and CHOP; 70% of patients in the A+CHP arm and 56% in the CHOP arm received 6 cycles of treatment. In ALK+ patients, 56% and 59% received 6 cycles of treatment in the A+CHP and CHOP arms, respectively; in ALK– patients 76% and 55% received 6 cycles, respectively.

A comparison between patients aged <60 years and those aged ≥60 years found generally similar rates of any-grade and grade ≥3 TEAEs between study arms and across age groups (Supplementary Table 6). Of note, a higher proportion of patients aged ≥60 years had any-grade febrile neutropenia (21%) compared with patients aged <60 years (7%), particularly in the A+CHP group (21% vs 13% in the CHOP group). In both treatment arms, serious TEAEs occurred at a higher rate in patients aged ≥60 years than in those aged <60 years (44% [*n* = 27] vs 24% [*n* = 24] in the A+CHP arm and 48% [*n* = 26] vs 29% [*n* = 29] in the CHOP arm).

Rates of treatment-emergent PN considered to be related to study treatment were 51% in the A+CHP arm and 47% in the CHOP arm (Supplementary Table 7); rates were 45% and 50%, respectively, among patients aged 18–40 and slightly higher in male versus female patients in both arms (55% vs 45%, and 48% vs 45%; Supplementary Table 8). Most treatment-emergent PN was grade 1 (60 [37.5%] and 63 [41%] patients in the A+CHP and CHOP arms, respectively) or grade 2 (21 patients [13%] and 17 [11%], respectively) with symptoms improved or completely resolved by the last visit. In the A+CHP arm, 53 patients (61%) had PN resolution, and 10 patients (11%) had improvement of PN events. In the CHOP arm, 59 patients (67%) had PN resolution, and 11 patients (13%) had improvement of PN. The median time to resolution or improvement of PN was 20.3 weeks (range 0–279) and 11.1 weeks (range 0–220) in patients in the A+CHP and CHOP arms, respectively. Rates of ongoing PN at the last visit were 39% and 33% in the A+CHP and CHOP arms, respectively, most of which were grade 1 (71% and 72%) or grade 2 (26% and 28%); only 1 patient in the A+CHP arm and none in the CHOP arm had ongoing grade ≥3 PN. No patients in the A+CHP arm discontinued treatment due to PN; 1 patient in the CHOP arm discontinued blinded study treatment due to PN.

Overall, 59 (37%) patients in the A+CHP arm and 60 (39%) in the CHOP arm experienced neutropenia. Use of G-CSF primary prophylaxis reduced the incidence of neutropenia in both study arms (Supplementary Table 9). Rates of grade ≥3 neutropenia without prophylaxis were 44% (48/109) in the A+CHP arm and 42% (49/117) in the CHOP arm, decreasing to 12% (6/51) and 14% (5/37), respectively, with prophylaxis. The rate of febrile neutropenia was similar in both treatment arms (13% and 10%); when using G-CSF prophylaxis, the rate decreased to 12% in the A+CHP and 3% in the CHOP arms. In the A+CHP arm, the effect of G-CSF prophylaxis was most marked in patients aged ≥60 years, in whom the majority of cases of febrile neutropenia occurred (Supplementary Table 9).

At data cut-off, 38 (24%) patients in the A+CHP arm and 49 (32%) in the CHOP arm had died. In the A+CHP arm, there were 24 (15%) disease-related deaths (e.g., sALCL [*n* = 15], cutaneous T-cell lymphoma [*n* = 1], PTCL [*n* = 1], and sepsis [*n* = 1]), as well as 10 non-disease-related deaths, and 4 of unknown relationship to disease (Supplementary Table 10). In the CHOP arm, there were 33 (21%) disease-related deaths (e.g., sALCL [*n* = 25], sepsis shock [*n* = 2], and lymphadenopathy [*n* = 1]), together with 8 non-disease-related deaths, and 8 of unknown relationship to disease (Supplementary Table 10).

**SUPPLEMENTARY TABLES AND FIGURES**

**Supplementary Table 1: List of IECs and IRBs**

| **Site Number** | **Institution** | **IEC/IRB Address** |
| --- | --- | --- |
| 10001 | Stanford University Research & Compliance Office | 3000 El Camino Real Five Palo Alto Square, 4th Floor Palo Alto, CA 94306 USA |
| 10002 | Washington University School of Medicine-Human Research Protection Office | 660 South Euclid Avenue Campus Box 8089 St. Louis, MO 63110 USA |
| 10003 | University of Texas MD Anderson Cancer Center | 7007 Bertner Avenue Unit 1637 Houston, TX 77030-4009 USA |
| 10004 | Western Institutional Review Board | 1019 39th Avenue SE Suite 120 Puyallup, WA 98374-2115 USA |
| 10005 | Institutional Review Board-Memorial Sloan Kettering Cancer Center | 1275 York Avenue New York, NY 10065 USA |
| 10006 | Dana Farber Cancer Institute Institutional Review Board | 450 Brookline Ave OS229 Boston, MA 02115 USA |
| 10007 | Thomas Jefferson University IRB | 1020 Locust Street M-34 Philadelphia, PA 19107 USA |
| 10008 | Western Institutional Review Board | 1019 39th Avenue SE Suite 120 Puyallup, WA 98374-2115 USA |
| 10009 | Institutional Review Board (Weill Cornell Medical College) | 1300 York Avenue Box 89 New York, NY 10065 USA |
| 10010 | Western Institutional Review Board | 1019 39th Avenue SE Suite 120 Puyallup, WA 98374-2115 USA |
| 10013 | Western Institutional Review Board | 1019 39th Avenue SE Suite 120 Puyallup, WA 98374-2115 USA |
| 10015 | Cleveland Clinic Foundation Institutional Review Board | 9500 Euclid Avenue OS-1 Cleveland, OH 44195 USA |
| 10017 | Western Institutional Review Board | 1019 39th Avenue SE Suite 120 Puyallup, WA 98374-2115 USA |
| 10018 | Western Institutional Review Board | 1019 39th Avenue SE Suite 120 Puyallup, WA 98374-2115 USA |
| 10019 | Western Institutional Review Board | 1019 39th Avenue SE Suite 120 Puyallup, WA 98374-2115 USA |
| 10020 | Columbia University Medical Center Institutional Review Board | 154 Haven Avenue 1st Floor New York, NY 10032 USA |
| 10024 | Brany IRB (Biomedical Research Alliance of New York) | 1981 Marcus Avenue Suite 210 Lake Success, NY 11042 USA |
| 10025 | Jewish Hospital Institutional Review Board | 4777 East Galbraith Road Cincinnati, OH 45236 USA |
| 10028 | Western Institutional Review Board | 1019 39th Avenue SE Suite 120 Puyallup, WA 98374-2115 USA |
| 10030 | Western Institutional Review Board | 1019 39th Avenue SE Suite 120 Puyallup, WA 98374-2115 USA |
| 10031 | Orlando Regional Healthcare System, Inc. IRB #1 - Orlando Regional Medical Center IRB | 1414 Kuhl Avenue MP#21 Orlando, FL 32806 USA |
| 10032 | University of Michigan Medical School IRBMED | 2800 Plymouth Road Building 520, Room 3214 Ann Arbor, MI 48109-2800 USA |
| 10035 | Western Institutional Review Board | 1019 39th Avenue SE Suite 120 Puyallup, WA 98374-2115 USA |
| 10037 | University of Virginia Institutional Review Board for Health Sciences Research (IRB-HSR) | One Morton Drive Suite 400, Box 5 Charlottesville, VA 22903 USA |
| 10040 | University of Kansas Medical Center - Human Subjects Committee | 3901 Rainbow Boulevard Kansas |
| 10041 | Western Institutional Review Board | 1019 39th Avenue SE Suite 120 Puyallup, WA 98374-2115 USA |
| 10045 | Western Institutional Review Board | 1019 39th Avenue SE Suite 120 Puyallup, WA 98374-2115 USA |
| 10054 | Brany IRB (Biomedical Research Alliance of New York) | 1981 Marcus Avenue Suite 210 Lake Success, NY 11042 USA |
| 10056 | Institutional Review Board-Memorial Sloan Kettering Cancer Center | 1275 York Avenue New York, NY 10065 USA |
| 10057 | Institutional Review Board-Memorial Sloan Kettering Cancer Center | 1275 York Avenue New York, NY 10065 USA |
| 11001 | UBC BCCA Research Ethics Board | 750 West Broadway Suite 1315 Vancouver, BC V5Z 1J3 Canada |
| 11002 | McGill University Health Center for Applied Ethics | 2155 Guy Street Room 231 Montreal, Quebec H3H 2R9 Canada |
| 11012 | McGill University Health Center for Applied Ethics | 2155 Guy Street Room 231 Montreal, Quebec H3H 2R9 Canada |
| 33001 | Comité de Protection des Personnes Ouest IV | Maison de la sante, 2eme etage 53 Chaussee de la Madeleine Nantes 44000 France |
| 33003 | Comité de Protection des Personnes Ouest IV | Maison de la sante, 2eme etage 53 Chaussee de la Madeleine Nantes 44000 France |
| 33004 | Comité de Protection des Personnes Ouest IV | Maison de la sante, 2eme etage 53 Chaussee de la Madeleine Nantes 44000 France |
| 33006 | Comité de Protection des Personnes Ouest IV | Maison de la sante, 2eme etage 53 Chaussee de la Madeleine Nantes 44000 France |
| 33007 | Comité de Protection des Personnes Ouest IV | Maison de la sante, 2eme etage 53 Chaussee de la Madeleine Nantes 44000 France |
| 33008 | Comité de Protection des Personnes Ouest IV | Maison de la sante, 2eme etage 53 Chaussee de la Madeleine Nantes 44000 France |
| 33009 | Comité de Protection des Personnes Ouest IV | Maison de la sante, 2eme etage 53 Chaussee de la Madeleine Nantes 44000 France |
| 33010 | Comité de Protection des Personnes Ouest IV | Maison de la sante, 2eme etage 53 Chaussee de la Madeleine Nantes 44000 France |
| 33011 | Comité de Protection des Personnes Ouest IV | Maison de la sante, 2eme etage 53 Chaussee de la Madeleine Nantes 44000 France |
| 33012 | Comité de Protection des Personnes Ouest IV | Maison de la sante, 2eme etage 53 Chaussee de la Madeleine Nantes 44000 France |
| 33013 | Comité de Protection des Personnes Ouest IV | Maison de la sante, 2eme etage 53 Chaussee de la Madeleine Nantes 44000 France |
| 33014 | Comité de Protection des Personnes Ouest IV | Maison de la sante, 2eme etage 53 Chaussee de la Madeleine Nantes 44000 France |
| 33016 | Comité de Protection des Personnes Ouest IV | Maison de la sante, 2eme etage 53 Chaussee de la Madeleine Nantes 44000 France |
| 34001 | Central: Hospital Universitario y Politecnico la Fe  Local: CEIC Area De Salud de Leon | Central: Secretaria del CIEC Torre A, planta 7º, Bulevar Sur, s/n Valencia 46026 Spain Local: Complejo Asistencial de Leon Edificio San Antonio Abad 2^ Planta, Chirurgia General II C/ Altos De Nava Leon 24017 Spain |
| 34002 | Central: Hospital Universitario y Politecnico la Fe  Local: Comite Etico de Investigacion Clinica, Servicio de Farmacologia Clinica | Central: Secretaria del CIEC Torre A, planta 7º, Bulevar Sur, s/n Valencia 46026 Spain Local: Hospital de la Santa Creu i Sant Pau, Avda Sant Antoni Ma Claret, 167 Barcelona 08025 Spain |
| 34004 | Central: Hospital Universitario y Politecnico la Fe | Central: Secretaria del CIEC Torre A, planta 7º, Bulevar Sur, s/n Valencia 46026 Spain |
| 34006 | Central: Hospital Universitario y Politecnico la Fe  Local: CEIC H. U Puerta de Hierro Majadahonda (Entrada por Laboratorio-Banco de Sangre) | Central: Secretaria del CIEC Torre A, planta 7º, Bulevar Sur, s/n Valencia 46026 Spain  Local: Planta 1 - peines 6/7 - C/Joaquin Rodrigo, 2 Majadahonda, Madrid 28222 Spain |
| 34007 | Central: Hospital Universitario y Politecnico la Fe  Local: CEIC Hospital Universitari de Bellvitge | Central: Secretaria del CIEC Torre A, planta 7º, Bulevar Sur, s/n Valencia 46026 Spain  Local: Edifici de la Unitat de Recerca, Feixa Llarga s/n L'Hospitalet de Llobregat Barcelona 08907 Spain |
| 34008 | Central: Hospital Universitario y Politecnico la Fe  Local: CEIC Hospital Universitario Gregorio Maranon | Central: Secretaria del CIEC Torre A, planta 7º, Bulevar Sur, s/n Valencia 46026 Spain  Local: Oficina Tecnica (CEIC AREA 1) Fundacion para la investigacion Edificio de la Investigacion, Planta Baja, Calle Maiquez 9 Madrid 28009 Spain |
| 34009 | Central: Hospital Universitario y Politecnico la Fe  Local: Area de Gestion de Proyectos-Unidad Administrativa CEIC - Instituto de Investigacion Hospital 12 de Octubre (I+12) | Central: Secretaria del CIEC Torre A, planta 7º, Bulevar Sur, s/n Valencia 46026 Spain  Local: Centro de Actividades Ambulatorias Bloque D. Planta 6, Avenida de Cordoba s/n Madrid 28041 Spain |
| 34011 | Central: Hospital Universitario y Politecnico la Fe  Local: Hospital Universitari Quiron Dexeus, Servicio de diagnostico por la Imagen | Central: Secretaria del CIEC Torre A, planta 7º, Bulevar Sur, s/n Valencia 46026 Spain  Local: Planta I, Calle Sabina Arana 5-19 Barcelona 08028 Spain |
| 34012 | Central: Hospital Universitario y Politecnico la Fe  Local: Comitè Etico de Investigacion Clinica | Central: Secretaria del CIEC Torre A, planta 7º, Bulevar Sur, s/n Valencia 46026 Spain  Local: Hospital Universitario de Salamanca Paseo de San Vicente, 58-182 Salamanca 37007 |
| 36002 | Egeszsegugyi Tudomanyos Tanacs - Klinikai Farmakologiai Etikai Bizottsaga | Szechenyi Istvan ter 7-8 Budapest 1051 Hungary |
| 36003 | Egeszsegugyi Tudomanyos Tanacs - Klinikai Farmakologiai Etikai Bizottsaga | Szechenyi Istvan ter 7-8 Budapest 1051 Hungary |
| 39001 | Central: Comitato Etico Milano AREA C- Azienda Ospedaliera Niguarda Ca' Granda | Central: Piazza Ospedale Maggiore, 3 Milano 20162 Italy |
| 39002 | Central: Comitato Etico Milano AREA C- Azienda Ospedaliera Niguarda Ca' Granda  Local: Comitato Etico dell'Azienda Socio Sanitaria Territoriale degli Spedali Civili di Brescia | Central: Piazza Ospedale Maggiore, 3 Milano 20162 Italy Local: Piazzale Spedali Civili, 1 Brescia 25123 Italy |
| 39003 | Central: Comitato Etico Milano AREA C- Azienda Ospedaliera Niguarda Ca' Granda  Local: Comitato Etico per la Sperimentazione Clinica dei Medicinali dell' Azienda Ospedaliera Universitaria Integrata di Verona | Central: Piazza Ospedale Maggiore, 3 Milano 20162 Italy Local: Piazzale A. Stefani, 1 Verona 37126 Italy |
| 39004 | Central: Comitato Etico Milano AREA C- Azienda Ospedaliera Niguarda Ca' Granda  Local: Comitato Etico della Fondazione IRCCS Ca' Granda Ospedale Maggiore Policlinico di Milano | Central: Piazza Ospedale Maggiore, 3 Milano 20162 Italy Local: Via Francesco Sforza 28 Milano 20122 Italy |
| 39005 | Central: Comitato Etico Milano AREA C- Azienda Ospedaliera Niguarda Ca' Granda  Local: Comitato Etico Regionale Liguria - Sezione N. 2 | Central: Piazza Ospedale Maggiore, 3 Milano 20162 Italy Local: Largo R. Benzi, 10 Genova 16132 Italy |
| 39007 | Central: Comitato Etico Milano AREA C- Azienda Ospedaliera Niguarda Ca' Granda  Local: Comitato Etico Interaziendale - Azienda Ospedaliera Città della Salute e della Scienza di Torino - A.O. Mauriziano - ASL TO | Central: Piazza Ospedale Maggiore, 3 Milano 20162 Italy Local: Corso Bramante, 88 Torino 10126 Italy |
| 39008 | Central: Comitato Etico Milano AREA C- Azienda Ospedaliera Niguarda Ca' Granda  Local: Azienda Ospedaliero Universitaria Policlinico Vittorio Emanuele | Central: Piazza Ospedale Maggiore, 3 Milano 20162 Italy Local: Via Santa Sofia, 78 Catania 95123 Italy |
| 39009 | Central: Comitato Etico Milano AREA C- Azienda Ospedaliera Niguarda Ca' Granda  Local: Comitato Etico Indipendente dell'Azienda Ospedaliera Universitaria - Policlinico S. Orsola Malpighi di Bologna | Central: Piazza Ospedale Maggiore, 3 Milano 20162 Italy Local: Via Pietro Albertoni, 15 Bologna 40138 Italy |
| 39011 | Central: Comitato Etico Milano AREA C- Azienda Ospedaliera Niguarda Ca' Granda  Local: Comitato Etico Indipendente, Istituto Clinico Humanitas - IRCCS | Central: Piazza Ospedale Maggiore, 3 Milano 20162 Italy Local: Via Manzoni, 56, Servizio Farmacia Milano 20089 Italy |
| 39012 | Central: Comitato Etico Milano AREA C- Azienda Ospedaliera Niguarda Ca' Granda  Local: Comitato etico della provincia di Bergamo | Central: Piazza Ospedale Maggiore, 3 Milano 20162 Italy Local: Piazza Organizzazione Mondiale della Sanità 1 Bergamo 24127 Italy |
| 39013 | Central: Comitato Etico Milano AREA C- Azienda Ospedaliera Niguarda Ca' Granda  Local: Comitato di Bioetica della Fondazione IRCCS Policlinico San Matteo di Pavia | Central: Piazza Ospedale Maggiore, 3 Milano 20162 Italy Local: Viale Golgi n° 19 Pavia 27100 Italy |
| 40004 | Comisa Nationala de Bioteca a Medicamentului si a Dispozitivelor | Sos. Stefan cal Mare nr. 19-21, sector 2, Incinta Spitalului Colentina, Pavilion K Bucuresti 020125 Romania |
| 40006 | Comisa Nationala de Bioteca a Medicamentului si a Dispozitivelor | Sos. Stefan cal Mare nr. 19-21, sector 2, Incinta Spitalului Colentina, Pavilion K Bucuresti 020125 Romania |
| 42001 | Central: Etická komise Fakultní nemocnice Brno  Local: Etická komise Fakultní nemocnice Hradec Králové | Central: Jihlavská 20 Brno, JIHORMORAVSKY KRAJ 625 00 Czech Republic Local: Sokolská 581 Hradec Králové, VYCHODOCESKY KRAJ 500 05 Czech Republic |
| 42002 | Central: Etická komise Fakultní nemocnice Brno | Central: Jihlavská 20 Brno, JIHORMORAVSKY KRAJ 625 00 Czech Republic |
| 42003 | Central: Etická komise Fakultní nemocnice Brno  Local: Fakultní Nemocnice Královské Vinohrady Etická Komise | Central: Jihlavská 20 Brno, JIHORMORAVSKY KRAJ 625 00 Czech Republic Local: Šrobárova 1150/50 Praha 10, 100 34 Czech Republic |
| 42004 | Central: Etická komise Fakultní nemocnice Brno  Local: Etická komise Všeobecné fakultní nemocnice v Praze | Central: Jihlavská 20 Brno, JIHORMORAVSKY KRAJ 625 00 Czech Republic Local: Na Bojišti 1 Praha 2, 128 08 Czech Republic |
| 42005 | Central: Etická komise Fakultní nemocnice Brno  Local: Etická komise Fakultní nemocnice Ostrava | Central: Jihlavská 20 Brno, JIHORMORAVSKY KRAJ 625 00 Czech Republic Local: 17 Listopadu 1790/5 Ostrava, SEVEROMORAVSKY KRAJ 708 52 Czech Republic |
| 44001 | Central: NRES Committee North West - Greater Manchester Central | Central: HRA NRES Centre - Manchester 3rd Floor, Barlow House 4 Minshull Street Manchester, England M1 3DZ United Kingdom |
| 44003 | Central: NRES Committee North West - Greater Manchester Central | Central: HRA NRES Centre - Manchester 3rd Floor, Barlow House 4 Minshull Street Manchester, England M1 3DZ United Kingdom |
| 44004 | Central: NRES Committee North West - Greater Manchester Central | Central: HRA NRES Centre - Manchester 3rd Floor, Barlow House 4 Minshull Street Manchester, England M1 3DZ United Kingdom |
| 44005 | Central: NRES Committee North West - Greater Manchester Central | Central: HRA NRES Centre - Manchester 3rd Floor, Barlow House 4 Minshull Street Manchester, England M1 3DZ United Kingdom |
| 44010 | Central: NRES Committee North West - Greater Manchester Central  Local: NHS Greater Glasgow & Clyde, Clinical Research & Development | Central: HRA NRES Centre - Manchester 3rd Floor, Barlow House 4 Minshull Street Manchester, England M1 3DZ United Kingdom Local: West Glasgow Ambulatory Care Hospital Dalnair Street Glasgow, G3 8SJ United Kingdom |
| 45001 | De Videnskabsetiske Komiteer For Region Midtjylland | Skottenborg 26 Viborg 8800 Denmark |
| 45002 | De Videnskabsetiske Komiteer For Region Midtjylland | Skottenborg 26 Viborg 8800 Denmark |
| 45004 | De Videnskabsetiske Komiteer For Region Midtjylland | Skottenborg 26 Viborg 8800 Denmark |
| 48001 | Komisja Bioetyczna przy Okregowej Izbie Lekarskiej | ulica Krupnicza 11a Kraków, MALOPOLSKIE 31-123 Poland |
| 48003 | Komisja Bioetyczna przy Okregowej Izbie Lekarskiej | ulica Krupnicza 11a Kraków, MALOPOLSKIE 31-123 Poland |
| 48004 | Komisja Bioetyczna przy Okregowej Izbie Lekarskiej | ulica Krupnicza 11a Kraków, MALOPOLSKIE 31-123 Poland |
| 49001 | Central: Ethik-Kommission der Georg-August-Universität Göttingen | Central: Von-Siebold-Straße 3 Göttingen 37075 Germany |
| 49002 | Central: Ethik-Kommission der Georg-August-Universität Göttingen  Local: Ethikkommission der Medizinischen Fakultät der Ludwig-Maximilians-Universität München | Central: Von-Siebold-Straße 3 Göttingen 37075 Germany Local: Pettenkoferstraße, 8 München 80366 Germany |
| 49003 | Central: Ethik-Kommission der Georg-August-Universität Göttingen  Local: Ethikkommission der Medizinischen Fakultät der Universität Erlangen-Nürnberg | Central: Von-Siebold-Straße 3 Göttingen 37075 Germany Local: Krankenhausstraße 12 Erlangen 91054 Germany |
| 49004 | Central: Ethik-Kommission der Georg-August-Universität Göttingen  Local: Ethikkommission der Friedrich-Schiller-Universität Jena an der Medizinischen Fakultät | Central: Von-Siebold-Straße 3 Göttingen 37075 Germany Local: Bachstraße 18, Gebaude 1 Jena 07740 Germany |
| 49005 | Central: Ethik-Kommission der Georg-August-Universität Göttingen  Local: Ärztekammer des Saarlandes -Ethikkommission | Central: Von-Siebold-Straße 3 Göttingen 37075 Germany Local: Faktoreistraße 4 Saarbrücken 66111 Germany |
| 49006 | Central: Ethik-Kommission der Georg-August-Universität Göttingen  Local: Ethikkommission der Medizinischen Fakultät der Universität Duisburg-Essen | Central: Von-Siebold-Straße 3 Göttingen 37075 Germany Local: Robert-Koch-Straße 9/11 Essen 45147 Germany |
| 49007 | Central: Ethik-Kommission der Georg-August-Universität Göttingen  Local: Ethikkommission der Medizinischen Fakultat der Universitat zu Koln | Central: Von-Siebold-Straße 3 Göttingen 37075 Germany Local: Kerpener Strasse 62, Gebaude 55 Koln 50937 Germany |
| 49008 | Central: Ethik-Kommission der Georg-August-Universität Göttingen  Local: Landesamt für Gesundheit und Soziales Berlin - EthikKommission des Landes Berlin | Central: Von-Siebold-Straße 3 Göttingen 37075 Germany Local: Fehrbelliner Platz 1 Berlin 10707 Germany |
| 49010 | Central: Ethik-Kommission der Georg-August-Universität Göttingen  Local: Ethik-Kommission der Landesärztekammer Hessen | Central: Von-Siebold-Straße 3 Göttingen 37075 Germany Local: Im Vogelsgesang 3 Frankfurt 60488 Germany |
| 49011 | Central: Ethik-Kommission der Georg-August-Universität Göttingen  Local: Ethik-Kommission I der Medizinischen Fakultat Heidelberg | Central: Von-Siebold-Straße 3 Göttingen 37075 Germany Local: Alte GlockengieBerei 11/1 Heidelberg 69115 Germany |
| 49014 | Central: Ethik-Kommission der Georg-August-Universität Göttingen  Local: Ethikkommission bei der Sachsischen Landesarztekammer | Central: Von-Siebold-Straße 3 Göttingen 37075 Germany Local: Schutzenhohe 16 Dresden 01099 Germany |
| 49015 | Central: Ethik-Kommission der Georg-August-Universität Göttingen  Local: Landesamt für Gesundheit und Soziales, Geschäftsstelle der Ethik-Kommission des Landes Berlin | Central: Von-Siebold-Straße 3 Göttingen 37075 Germany Local: Fehrbelliner Platz 1 Berlin 10707 Germany |
| 61001 | Hunter New England Human Research Ethics Committee | Hunter New England Research Ethics and Governance Unit Hunter New England Local Health District, Lookout Road New Lambton, NSW 2305 Australia |
| 61004 | Hunter New England Human Research Ethics Committee | Hunter New England Research Ethics and Governance Unit Hunter New England Local Health District, Lookout Road New Lambton, NSW 2305 Australia |
| 61005 | Hunter New England Human Research Ethics Committee | Hunter New England Research Ethics and Governance Unit Hunter New England Local Health District, Lookout Road New Lambton, NSW 2305 Australia |
| 61006 | Hunter New England Human Research Ethics Committee | Hunter New England Research Ethics and Governance Unit Hunter New England Local Health District, Lookout Road New Lambton, NSW 2305 Australia |
| 61007 | Hunter New England Human Research Ethics Committee | Hunter New England Research Ethics and Governance Unit Hunter New England Local Health District, Lookout Road New Lambton, NSW 2305 Australia |
| 61010 | Hunter New England Human Research Ethics Committee | Hunter New England Research Ethics and Governance Unit Hunter New England Local Health District, Lookout Road New Lambton, NSW 2305 Australia |
| 61018/61008^a^ | Bellberry Human Research Ethics Committee | 129 Glen Osmond Road  Eastwood, Adelaide SA 5065 Australia |
| 81001 | Tohoku University Hospital Institutional Review Board | 1-1 Seiryo-machi, Aoba-ku Sendai-city 980-8574 Japan |
| 81002 | The Cancer Institute Hospital of JFCR IRB | 3-8-31 Ariake, Koto-ku Tokyo 135-8550 Japan |
| 81003 | National Cancer Center Hospital Institutional Review Board | 5-1-1 Tsukiji, Chuo-ku Tokyo 104-0045 Japan |
| 81005 | National Hospital Organization Kyushu Cancer Center IRB | 3-1-1, Notame, Minami-ku Fukuoka 811-1395 Japan |
| 81006 | National Cancer Center Hospital Institutional Review Board | 5-1-1 Tsukiji, Chuo-ku Tokyo 104-0045 Japan |
| 81007 | University Hospital, Kyoto Prefectural University of Medicine Institutional Review Board | 465 Kajii-cho, Kawaramachi-hirokoji, Kamigyo-ku Kyoto-city 602-8566 Japan |
| 81008 | Okayama University Hospital Institutional Review Board | 2-5-1 Shikata-cho, Kita-ku Okayama 700-8558 Japan |
| 81009 | Kyushu University Hospital IRB | 3-1-1, Maidashi, Higashi-ku Fukuoka 812-8582 Japan |
| 81010 | Tokai University Hospital Institutional Review Board | 143, Shimokasuya Isehara-shi, Kanagawa 259-1193 Japan |
| 81011 | Aichi Cancer Center Hospital Institutional Review Board | 1-1 Kanokoden, Chikusa-ku Nagoya 464-8681 Japan |
| 81013 | National Hospital Organization Nagoya Medical Center IRB | 4-1-1 Sannomaru, Naka-ku Nagoya-city 460-0001 Japan |
| 81014 | Osaka University Hospital Institutional Review Board | 2-15, Yamadaoka Suita, Osaka 565-0871 Japan |
| 82001 | Asan Medical Center | 88 Olympic-ro 43-gil, Songpa-gu Seoul 05505 South Korea |
| 82003 | Seoul National University Bundang Hospital | 82 Gumi-Ro 173 Beon-Gil, Bundang-gu Seongnam-si, Gyeonggi 13620 South Korea |
| 82004 | Severance Hospital Institutional Review Board (Yonsei University College of Medicine) | 50-1 Yonsei-ro, Seodaemun-gu Seoul 03722 South Korea |
| 82006 | Seoul National University Hospital Institutional Review Board | 101 Daehak-ro, Jongno-gu Seoul 03080 South Korea |
| 82007 | Samsung Medical Center Institutional Review Board | Irwon-ro 81, Gangnam-gu Seoul 06351 South Korea |
| 82008 | Keimyung University Dongsan Medical Center | 56 Dalseong-ro, Jung-gu Daegu 41931 South Korea |
| 82010 | Chungnam National University Hospital Institutional Review Board | 282 Munhwa-ro, Jung-gu Daejeon 35015 South Korea |
| 88601 | Chang Gung Medical Foundation Institutional Review Board | 199 Tung Hwa North Road Taipei 10507 Taiwan |
| 88602 | Chang Gung Medical Foundation Institutional Review Board | 199 Tung Hwa North Road Taipei 10507 Taiwan |
| 88603 | Research Ethics Committee China Medical University & Hospital | 2 Yude Road, Number 1 Medical Building 9F, North District Taichung City 404 Taiwan |
| 97201 | Hadassah Medical Center, Helsinki Committee | Kiryat Hadassah  Ein Kerem Jerusalem 9112001 Israel |
| 97202 | Rambam Medical Center, Institutional Review Board | 6 HaAliya HaShniya St PO Box 9602 Haifa 3109601 Israel |
| 97203 | Soroka Medical Center Institutional Review Board/Ethics Committee | Soroka Medical Center PO Box 151 Beer Sheva 84101 Israel |
| 97204 | Rabin Medical Center | 39 Jabotinsky St. Petah Tikva 49100 Israel |
| 97205 | The Tel Aviv Sourasky Medical Center | 6 Weizmann St. Tel-Aviv 64239 Israel |

^a^Site 61018 did not enrol any patients. A patient was enrolled at 61008 and then transferred to 61018

*IEC* independent ethics committee, *IRB* institutional review board.

**Supplementary Table 2.** Baseline characteristics of patients with sALCL by ALK− and ALK+ subgroups in ECHELON-2 (intent-to-treat population, *N =* 316).

|  | **A+CHP** | | | **CHOP** | | |
| --- | --- | --- | --- | --- | --- | --- |
|  | **ALK+**  ***n* = 49** | **ALK−**  ***n* = 113** | **Overall**  ***n* = 162** | **ALK+**  ***n* = 49** | **ALK−**  ***n* = 105** | **Overall**  ***n* = 154** |
| Age |  |  |  |  |  |  |
| <60 years | 41 (84) | 60 (53) | 101 (62) | 40 (82) | 60 (57) | 100 (65) |
| ≥60 years | 8 (16) | 53 (47) | 61 (38) | 9 (18) | 45 (43) | 54 (35) |
| <40 years | 17 (35) | 15 (13) | 32 (20) | 21 (43) | 15 (14) | 36 (23) |
| ≥40 years | 32 (65) | 98 (87) | 130 (80) | 28 (57) | 90 (86) | 118 (77) |
| ECOG PS |  |  |  |  |  |  |
| 0 or 1 | 24 (49) | 96 (85) | 120 (74) | 28 (57) | 86 (82) | 114 (74) |
| 2 | 25 (51) | 16 (14) | 41 (25) | 21 (43) | 19 (18) | 40 (26) |
| Missing | 0 | 1 (<1) | 1 (<1) | 0 | 0 | 0 |
| IPI score |  |  |  |  |  |  |
| 0–1 | 1 (2) | 40 (35) | 41 (25) | 0 | 32 (30) | 32 (21) |
| 2–3 | 43 (88) | 52 (46) | 95 (59) | 43 (88) | 57 (55) | 100 (65) |
| 4–5 | 5 (10) | 21 (19) | 26 (16) | 6 (12) | 16 (15) | 22 (14) |
| Disease stage at diagnosis |  |  |  |  |  |  |
| I or II | 7 (14) | 27 (24) | 34 (21) | 1 (2) | 33 (32) | 34 (22) |
| III or IV | 42 (85) | 86 (76) | 128 (79) | 48 (98) | 72 (69) | 120 (78) |
| Serum LDH per local laboratory |  |  |  |  |  |  |
| ≤1 x ULN | 27 (55) | 60 (53) | 87 (54) | 26 (53) | 46 (44) | 72 (47) |
| >1 x ULN | 22 (45) | 53 (47) | 75 (46) | 23 (47) | 59 (58) | 82 (53) |
| Extranodal disease involvement |  |  |  |  |  |  |
| ≤1 site | 20 (41) | 74 (65) | 94 (58) | 23 (47) | 72 (69) | 95 (62) |
| >1 site | 29 (59) | 39 (35) | 68 (42) | 26 (53) | 33 (31) | 59 (38) |
| Baseline B symptoms^a^ |  |  |  |  |  |  |
| Yes | 20 (41) | 24 (21) | 44 (27) | 21 (43) | 33 (31) | 54 (35) |

^a^If a patient has at least one of the three B-symptoms (fever, drenching night sweats, and loss of more than 10% of body weight over 6 months) at baseline, the patients are counted in baseline B-symptoms. *A+CHP* brentuximab vedotin plus cyclophosphamide, doxorubicin, and prednisone, *ALK* anaplastic lymphoma kinase, *CHOP* cyclophosphamide, doxorubicin, vincristine, and prednisone, *ECOG PS* Eastern Cooperative Oncology Group performance status, *IPI*, International Prognostic Index, *LDH*, lactate dehydrogenase, *sALCL*, systemic anaplastic large cell lymphoma, *ULN*, upper limit of normal.

**Supplementary Table 3.** Summary of patients with relapsed or refractory disease after end of treatment per investigator (intent-to-treat population, *N =* 316).

| **Patients, *n* (%)** | **A+CHP *n =* 162** | **CHOP *n =* 154** |
| --- | --- | --- |
| Patients with relapsed/recurrent disease^a^ | 29 (18) | 22 (14) |
| Patients with refractory disease^b^ | 16 (10) | 42 (27) |
| Patients with relapsed/recurrent disease who received subsequent anti-cancer therapy | 21 (13) | 21 (14) |
| Disease relapse/recurrence before the first subsequent anti-cancer therapy | 21 (100) | 20 (95) |
| Disease relapse/recurrence after the first subsequent anti-cancer therapy | 0 (0) | 1 (5) |
| Patients’ survival status after the first relapse/recurrence |  |  |
| Alive | 15 (9) | 14 (9) |
| Dead | 14 (9) | 8 (5) |

^a^Relapsed/recurrent disease is defined as disease progression after achieving a complete response at end of treatment per investigator. ^b^Refractory disease is defined as having not achieved complete or partial response at end of treatment per investigator.

*A+CHP* brentuximab vedotin plus cyclophosphamide, doxorubicin, and prednisone, *CHOP* cyclophosphamide, doxorubicin, vincristine, and prednisone, *sALCL* systemic anaplastic large cell lymphoma.

**Supplementary Table 4.** Summary of subsequent anti-cancer therapies by treatment group (intent-to-treat population, *N =* 316).

| **Patients, *n* (%)** | **A+CHP *n =* 162** | **CHOP**  ***n =* 154** |
| --- | --- | --- |
| Patients who received subsequent new anti-cancer therapy | 35 (22) | 58 (38) |
| Systemic therapy for residual or progressive disease | 33 (20) | 58 (38) |
| Palliative radiation | 9 (6) | 5 (3) |
| Consolidative treatment received, n (%) | 48 (30) | 23 (15) |
| Radiotherapy | 14 (9) | 4 (3) |
| Stem cell transplant (autologous^a^) | 37 (23) | 20 (13) |
| Anti-cancer treatments received^b^ |  |  |
| Brentuximab vedotin-containing | 18 (11) | 39 (25) |
| Monotherapy | 16 (10) | 34 (22) |
| As part of combination therapy | 4 (2)^c^ | 5 (3) |
| ICE^d^ | 2 (1) | 7 (5) |
| Romidepsin | 4 (2) | 3 (2) |
| DHAP^e^ | 1 (1) | 6 (4) |
| ESHAP | 1 (1) | 4 (3) |
| Bendamustine | 2 (1) | 2 (1) |
| Methotrexate | 2 (1) | 2 (1) |
| Pralatrexate | 3 (2) | 1 (1) |
| Radiotherapy | 4 (2) | 2 (1) |
| Rituximab | 1 (1) | 1 (1) |
| Vinblastine | 2 (1) | 0 |

^a^No patients received allogeneic SCT.

^b^Occurring in >1 patient in either treatment arm.

^c^Two patients received brentuximab vedotin both as monotherapy and as part of combination therapy.

^d^Includes ICE chemotherapy in 2 patients in the CHOP arm.

^e^Includes DHAP chemotherapy in 2 patients in the CHOP arm.

*DHAP* dexamethasone, high-dose cytarabine, and cisplatin, *ESHAP* etoposide, cytarabine, cisplatin, and methylprednisolone, *ICE* ifosfamide, carboplatin, and etoposide, *SCT*, stem cell transplant.

**Supplementary Table 5.** Summary of treatment-emergent adverse events in patients with sALCL (safety population, *N =* 314).

| **Patients, *n* (%)** | **A+CHP *n =* 160** | **CHOP *n =* 154** |
| --- | --- | --- |
| Any TEAE | 159 (99) | 150 (97) |
| Grade ≥3 TEAE | 94 (59) | 98 (64) |
| Serious TEAE | 51 (32) | 55 (36) |
| TEAE leading to treatment discontinuation | 6 (4) | 14 (9) |
|  |  |  |
| Most common grade ≥3 TEAE^a^ |  |  |
| Neutropenia | 54 (34) | 53 (34) |
| Febrile neutropenia | 20 (13) | 16 (10) |
| Anemia | 17 (11) | 14 (9) |
| Leukopenia | 9 (6) | 9 (6) |

^a^TEAEs occurring in ≥5% of patients in either treatment arm.
*A+CHP* brentuximab vedotin plus cyclophosphamide, doxorubicin, and prednisone, *CHOP* cyclophosphamide, doxorubicin, vincristine, and prednisone, *sALCL* systemic anaplastic large cell lymphoma, *TEAE* treatment-emergent adverse event.

**Supplementary Table 6.** Summary of treatment-emergent adverse events in patients with sALCL by age group (safety population, *N =* 314).

|  | **A+CHP** | | **CHOP** | | |
| --- | --- | --- | --- | --- | --- |
| **Any event, *n* (%)** | **Age <60 years *n =* 99** | **Age ≥60 years *n =* 61** | | **Age <60 years *n =* 100** | **Age ≥60 years *n =* 54** |
| TEAE | 98 (99) | 61 (100) | 98 (98) | | 52 (96) |
| Neutropenia | 40 (40) | 19 (31) | 35 (35) | | 24 (44) |
| Febrile neutropenia | 7 (7) | 13 (21) | 9 (9) | | 7 (13) |
| Related | 86 (87) | 57 (93) | 84 (84) | | 45 (83) |
| Grade ≥3 | 54 (55) | 40 (66) | 60 (60) | | 38 (70) |
| Serious TEAE | 24 (24) | 27 (44) | 29 (29) | | 26 (48) |
| Discontinuation | 1 (1) | 5 (8) | 9 (9) | | 5 (9) |
| Fatal^a^ | 1 (1) | 3 (5) | 7 (7) | | 8 (15) |
| Febrile Neutropenia | 0 | 0 | 1 (1) | | 0 |
| Hydrocephalus | 0 | 0 | 1 (1) | | 0 |
| Multiple organ dysfunction syndrome | 0 | 0 | 1 (1) | | 0 |
| Sepsis | 0 | 0 | 2 (2) | | 0 |
| Cardiac arrest | 0 | 1 (2) | 1 (1) | | 0 |
| Cardiac failure acute | 0 | 0 | 1 (1) | | 0 |
| Ventricular fibrillation | 0 | 1 (2) | 0 | | 0 |
| ALCL T- and null-cell types | 0 | 0 | 2 (2) | | 6 (11) |
| Acute kidney injury | 1 (1) | 0 | 0 | | 0 |
| Other^b^ | 0 | 0 | 0 | | 1 (2) |
| Septic shock | 0 | 0 | 0 | | 1 (2) |
| Pulmonary cavitation | 0 | 1 (2) | 0 | | 0 |

^a^Two patients had 2 fatal events each. ^b^Preferred term, death; primary system organ class, general disorders, and administration site conditions.

*A+CHP* brentuximab vedotin plus cyclophosphamide, doxorubicin, and prednisone, *CHOP* cyclophosphamide, doxorubicin, vincristine, and prednisone, *sALCL* systemic anaplastic large cell lymphoma, *TEAE* treatment-emergent adverse event.

**Supplementary Table 7.** Summary of treatment-emergent peripheral neuropathy in patients with sALCL (safety population, *N =* 314).

|  | **A+CHP** | **CHOP** |
| --- | --- | --- |
| **Outcomes in all patients** | ***n =* 160** | ***n =* 154** |
| Patients with treatment-emergent PN, *n* (%) | 87 (54) | 88 (57) |
| Treatment-related | 82 (51) | 73 (47) |
| Grade 1^a^ | 60 (38) | 63 (41) |
| Grade 2^a^ | 21 (13) | 17 (11) |
| Grade 3^a^ | 5 (3) | 8 (5) |
| Grade 4^a^ | 1 (1) | 0 |
| Patients discontinuing blinded study treatment due to PN, *n* (%) | 0 | 1 (<1) |
| **Outcomes in patients with treatment-emergent PN** | ***n =* 87** | ***n =* 88** |
| Patients with resolution/improvement of PN events, *n* (%)^b^ |  |  |
| Resolution | 53 (61) | 59 (67) |
| Improvement | 10 (11) | 11 (13) |
| Median time to resolution/improvement of PN events, weeks (range)^c^ | 20.3 (0–279) | 11.1 (0–220) |
| Patients with ongoing PN events at last visit, *n* (%) | 34 (39) | 29 (33) |
| Grade 1 | 24 (28) | 21 (24) |
| Grade 2 | 9 (10) | 8 (9) |
| Grade 3 | 1 (1) | 0 |

^a^Worst severity. ^b^Resolution was defined as resolved/recovered with or without sequelae; or return to baseline or lower severity as of the latest assessment for pre-existing events. Improvement was defined as resolution or a decrease by at least one grade from the worst grade with no higher grade thereafter. Patients with improvement in any event at last follow-up were those with at least one improved event and the date of improvement was before last follow-up date. Patients with all events resolved were excluded. ^c^Time to resolution is used for resolved events and time to improvement is used for unresolved for improved events.
*A+CHP* brentuximab vedotin plus cyclophosphamide, doxorubicin, and prednisone, *CHOP* cyclophosphamide, doxorubicin, vincristine, and prednisone, *PN* peripheral neuropathy, *sALCL* systemic anaplastic large cell lymphoma.

**Supplementary Table 8.** Summary of treatment-emergent peripheral neuropathy in patients with sALCL, by sex and in the AYA subgroup (safety population, *N =* 314).

|  | **A+CHP**  **(*N* = 160)** | | | **CHOP**  **(*N* = 154)** | | |
| --- | --- | --- | --- | --- | --- | --- |
|  | AYA  (*n* = 31^a^) | Male (*n* = 94) | Female (*n* = 66) | AYA  (*n* = 36) | Male (*n* = 110) | Female (*n* = 44) |
| Patients with treatment-emergent PN, *n* (%) | 14 (45) | 55 (59) | 32 (48) | 20 (56) | 65 (59) | 23 (52) |
| Treatment-related | 14 (45) | 52 (55) | 30 (45) | 18 (50) | 53 (48) | 20 (45) |
| Grade 1^b^ | 12 (39) | 39 (41) | 21 (32) | 19 (53) | 47 (43) | 16 (36) |
| Grade 2^b^ | 1 (3) | 11 (12) | 10 (15) | 0 | 12 (11) | 5 (11) |
| Grade 3^b^ | 1 (3) | 4 (4) | 1 (2) | 1 (3) | 6 (5) | 2 (5) |
| Grade 4^b^ | 0 | 1 (1) | 0 | 0 | 0 | 0 |
| Patients discontinuing blinded study treatment due to PN, *n* (%) | 0 | 0 | 0 | 0 | 1 (1) | 0 |
| Subjects with ongoing peripheral neuropathy adverse events at last follow-up, *n* (%)^c^ | 3 (21) | 17 (31) | 17 (53) | 2 (10) | 23 (35) | 6 (26) |
| Grade 1^d^ | 3 (21) | 14 (25) | 10 (31) | 2 (10) | 17 (26) | 4 (17) |
| Grade 2^d^ | 0 | 2 (4) | 7 (22) | 0 | 6 (9) | 2 (9) |
| Grade 3^d^ | 0 | 1 (2) | 0 | 0 | 0 | 0 |
| Grade 4^c^ | 0 | 0 | 0 | 0 | 0 | 0 |

^a^Of the 32 enrolled patients aged <40 years, 1 patient did not receive A+CHP, and therefore could not be included in the safety population. ^b^Worst severity. ^c^Percentages are calculated out of the patients with treatment-emergent PN. ^d^Maximum severity at last follow-up.
*A+CHP* brentuximab vedotin plus cyclophosphamide, doxorubicin, and prednisone, *AYA* aged <40 years (18–40 years), *CHOP* cyclophosphamide, doxorubicin, vincristine, and prednisone, *PN* peripheral neuropathy, *sALCL* systemic anaplastic large cell lymphoma.

**Supplementary Table 9.** Summary of febrile neutropenia and neutropenia by use of G-CSF primary prophylaxis in patients with sALCL (safety population, *N =* 314).

|  | **A+CHP** | | | **CHOP** | | |
| --- | --- | --- | --- | --- | --- | --- |
| **Patients, *n* (%)** | **Overall** | **No G-CSF PP** | **G-CSF  PP** | **Overall** | **No G-CSF PP** | **G-CSF PP** |
| **Overall** | ***n =* 160** | ***n =* 109** | ***n =* 51** | ***n =* 154** | ***n =* 117** | ***n =* 37** |
| On-study febrile neutropenia | 20 (13) | 14 (13) | 6 (12) | 16 (10) | 15 (13) | 1 (3) |
| Neutropenia | 59 (37) | 51 (47) | 8 (16) | 60 (39) | 53 (45) | 7 (19) |
| Grade ≥3 neutropenia | 54 (34) | 48 (44) | 6 (12) | 54 (35) | 49 (42) | 5 (14) |
| Grade ≥4 neutropenia | 31 (19) | 27 (25) | 4 (8) | 35 (23) | 31 (26) | 4 (11) |
| Grade ≥3 infections and infestations (SOC) | 23 (14) | 18 (17) | 5 (10) | 17 (11) | 14 (12) | 3 (8) |
|  |  |  |  |  |  |  |
| **Age <60 years** | ***n =* 99** | ***n =* 75** | ***n =* 24** | ***n =* 100** | ***n =* 83** | ***n =* 17** |
| On-study febrile neutropenia | 7 (7) | 5 (7) | 2 (8) | 9 (9) | 8 (10) | 1 (6) |
| Neutropenia | 40 (40) | 38 (51) | 2 (8) | 35 (35) | 33 (40) | 2 (12) |
| Grade ≥3 neutropenia | 36 (36) | 35 (47) | 1 (4) | 31 (31) | 30 (36) | 1 (6) |
| Grade ≥4 neutropenia | 19 (19) | 19 (25) | 0 | 19 (19) | 18 (22) | 1 (6) |
| Grade ≥3 infections and infestations (SOC) | 15 (15) | 11 (15) | 4 (17) | 6 (6) | 5 (6) | 1 (6) |
|  |  |  |  |  |  |  |
| **Age ≥60 years** | ***n =* 61** | ***n =* 34** | ***n =* 27** | ***n =* 54** | ***n =* 34** | ***n =* 20** |
| On-study febrile neutropenia | 13 (21) | 9 (26) | 4 (15) | 7 (13) | 7 (21) | 0 |
| Neutropenia | 19 (31) | 13 (38) | 6 (22) | 25 (46) | 20 (59) | 5 (25) |
| Grade ≥3 neutropenia | 18 (30) | 13 (38) | 5 (19) | 23 (43) | 19 (56) | 4 (20) |
| Grade ≥4 neutropenia | 12 (20) | 8 (24) | 4 (15) | 16 (30) | 13 (38) | 3 (15) |
| Grade ≥3 infections and infestations (SOC) | 8 (13) | 7 (21) | 1 (4) | 11 (20) | 9 (26) | 2 (10) |

*A+CHP* brentuximab vedotin plus cyclophosphamide, doxorubicin, and prednisone, *CHOP* cyclophosphamide, doxorubicin, vincristine, and prednisone, *G-CSF* granulocyte colony-stimulating factor, *PP* primary prophylaxis, *sALCL* systemic anaplastic large cell lymphoma, *SOC* system organ class.

**Supplementary Table 10.** Summary of the causes of death in patients with sALCL (safety population, *N =* 314).

|  | **A+CHP (*N* = 160)** | **CHOP (*N* = 154)** | **Total (*N* = 314)** |
| --- | --- | --- | --- |
| **Overall, *n* (%)** | **38 (24)** | **49 (32)** | **87 (28)** |
| Disease-related deaths | 24 (15) | 33 (21) | 57 (18) |
| Acute kidney injury | 1 (1) | 0 | 1 (0) |
| Aspiration | 1 (1) | 0 | 1 (0) |
| Febrile neutropenia/sepsis^a^ | 1 (1) | 4 (3) | 5 (2) |
| Gastrointestinal haemorrhage | 1 (1) | 0 | 1 (0) |
| Hydrocephalus | 0 | 1 (1) | 1 (0) |
| Lung neoplasm (malignant) | 1 (1) | 0 | 1 (0) |
| Lymphoma progression^b^ | 18 (11) | 28 (18) | 46 (15) |
| Respiratory failure | 1 (1) | 0 | 1 (0) |
| Non-disease-related deaths | 10 (6) | 8 (5) | 18 (6) |
| Cardiac arrest | 1 (1) | 0 | 1 (0) |
| Cardiogenic shock | 1 (1) | 0 | 1 (0) |
| Complications of bone marrow transplant | 0 | 1 (1) | 1 (0) |
| Cytokine release syndrome | 1 (1) | 0 | 1 (0) |
| Other malignancies^c^ | 2 (1) | 2 (1) | 4 (1) |
| Pancreatitis acute | 0 | 1 (1) | 1 (0) |
| Pneumonitis^d^ | 1 (1) | 1 (1) | 2 (1) |
| Pulmonary cavitation | 1 (1) | 0 | 1 (0) |
| Sepsis^a^ | 1 (1) | 2 (1) | 3 (1) |
| Ventricular fibrillation | 1 (1) | 0 | 1 (0) |
| Unknown relationship | 5 (3) | 9 (6) | 14 (4) |
|  | | | |
| **Deaths within the safety analysis^e^ period, *n* (%)** | **4 (3)** | **12 (8)** | **16 (5)** |
| Disease-related deaths | 1 (1) | 7 (5) | 8 (3) |
| Non-disease-related deaths | 3 (2) | 1 (1) | 4 (1) |
| Cardiac arrest | 1 (1) | 0 | 1 (0) |
| Pulmonary cavitation | 1 (1) | 0 | 1 (0) |
| Sepsis^a^ | 0 | 1 (1) | 1 (0) |
| Ventricular fibrillation | 1 (1) | 0 | 1 (0) |
| Unknown relationship | 0 | 4 (3) | 4 (1) |
|  | | | |
| **Deaths after the safety analysis period, *n* (%)** | **34 (21)** | **37 (24)** | **71 (23)** |
| Disease-related deaths | 23 (14) | 26 (17) | 49 (16) |
| Non-disease-related deaths | 7 (4) | 7 (5) | 14 (4) |
| Cardiogenic shock | 1 (1) | 0 | 1 (0) |
| Complications of bone marrow transplant | 0 | 1 (1) | 1 (0) |
| Cytokine release syndrome | 1 (1) | 0 | 1 (0) |
| Other malignancies^c^ | 2 (1) | 2 (1) | 4 (1) |
| Pancreatitis acute | 0 | 1 (1) | 1 (0) |
| Pneumonitis^d^ | 1 (1) | 1 (1) | 2 (1) |
| Sepsis^a^ | 1 (1) | 1 (1) | 2 (1) |
| Unknown relationship | 5 (3) | 5 (3) | 10 (3) |

^a^Includes sepsis and septic shock. ^b^Includes disease progression, lymphadenopathy, metastases to bone, metastases to central nervous system, peripheral T-cell lymphoma unspecified, ALCL (T-cell and null types), and cutaneous T-cell lymphoma. ^c^Includes acute myeloid leukemia, adenocarcinoma, cutaneous T-cell lymphoma, and lung neoplasm malignant. ^d^Includes pneumonitis and pneumonia. ^e^The safety analysis period is defined as day 1 up to 30 days after the last dose of any drug in the regimen.
*A+CHP* brentuximab vedotin plus cyclophosphamide, doxorubicin, and prednisone, *ALCL* anaplastic large cell lymphoma, *CHOP* cyclophosphamide, doxorubicin, vincristine, and prednisone, *sALCL* systemic anaplastic large cell lymphoma

**Supplementary Figure 1.** Patient flow for patients with systemic anaplastic large cell lymphoma.

**
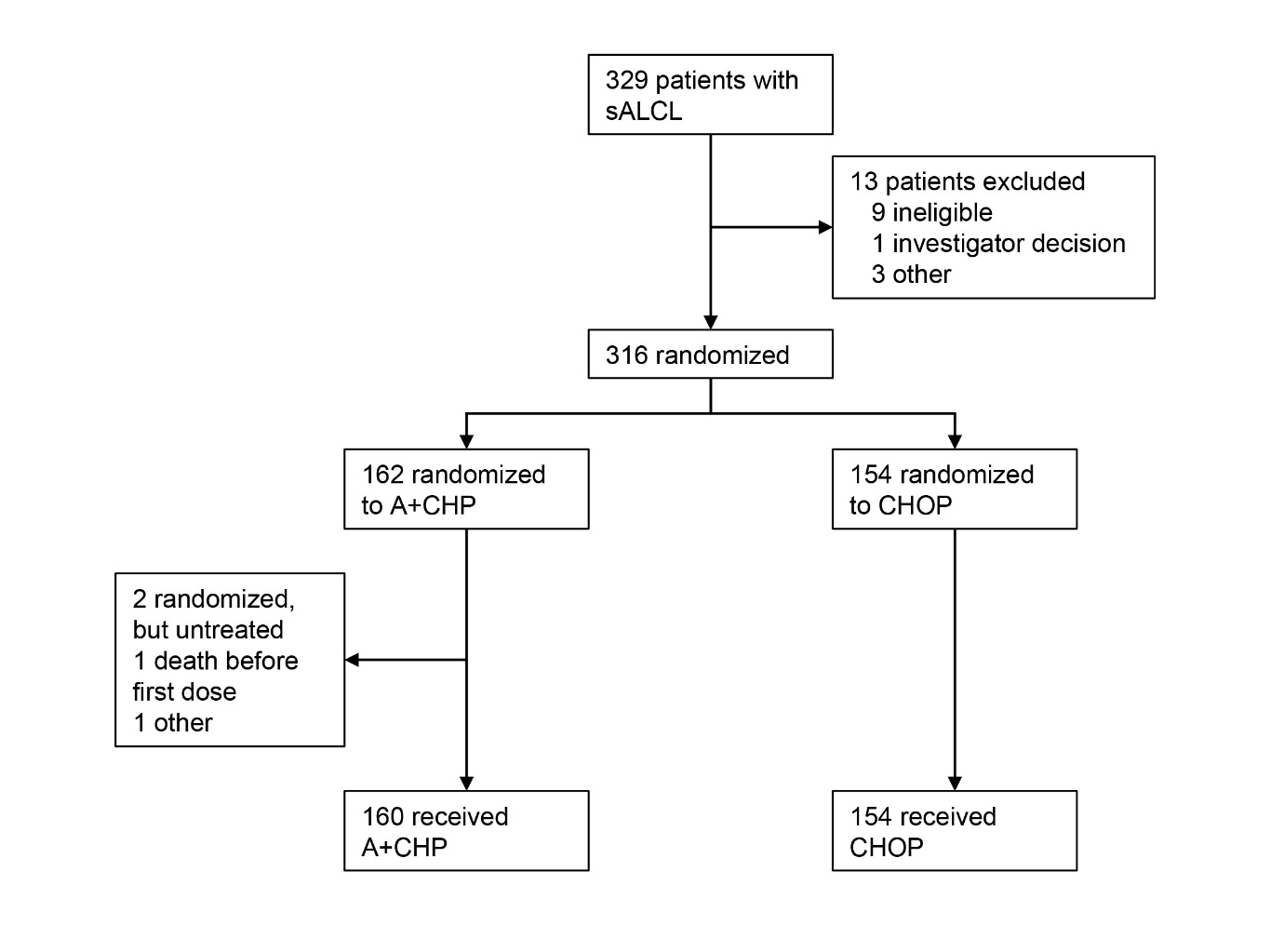
**

*A+CHP* brentuximab vedotin plus cyclophosphamide, doxorubicin, and prednisone, *CHOP* cyclophosphamide, doxorubicin, vincristine, and prednisone, *sALCL* systemic anaplastic large cell lymphoma.

**Supplementary Figure 2.** Forest plot of hazard ratios for progression-free survival per investigator according to baseline characteristics in (A) patients with sALCL and in (B) ALK+ and (C) ALK– sALCL subgroups (intent-to-treat population, *N =* 316).

**
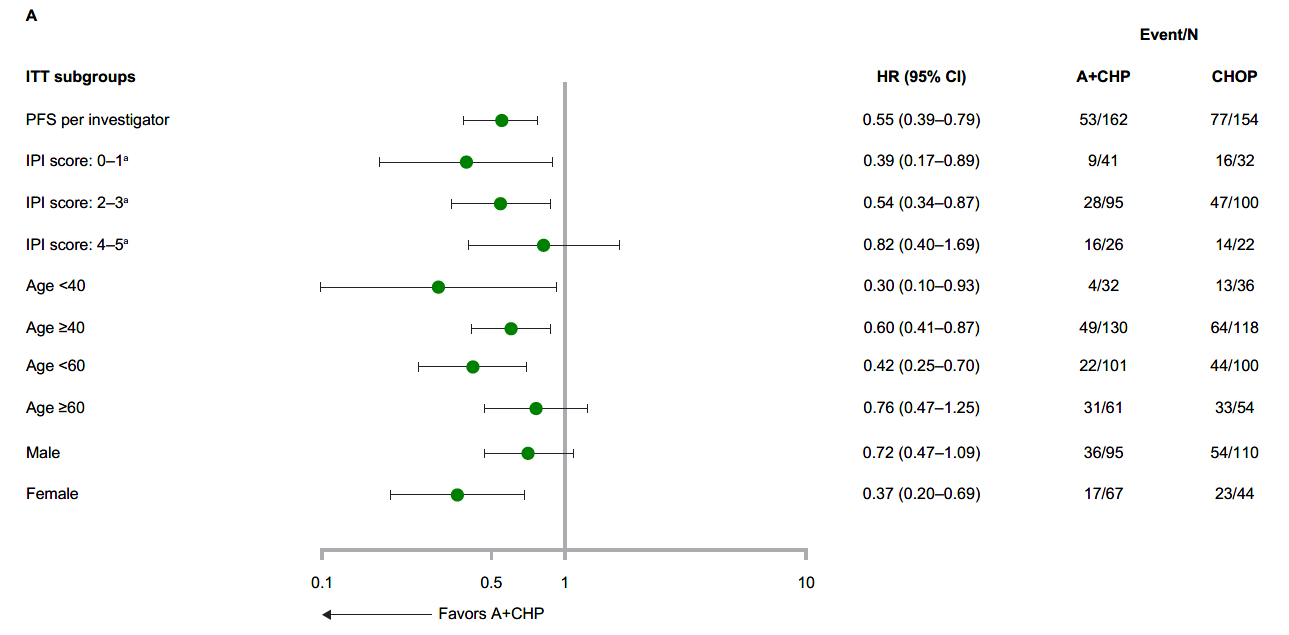
**

**
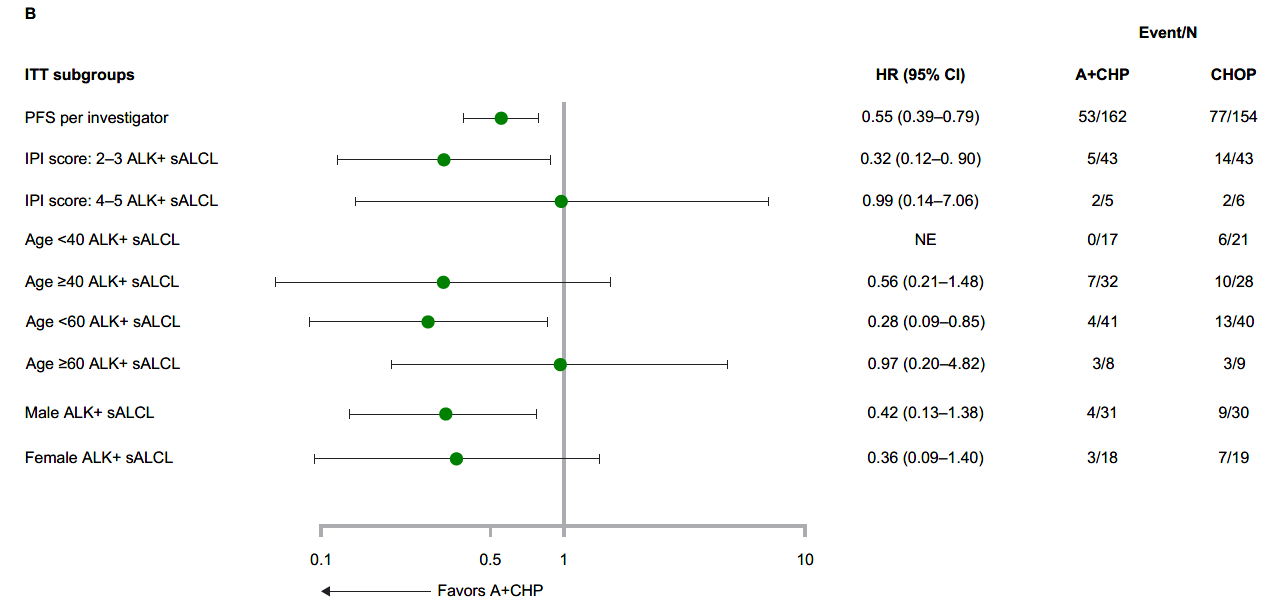
**

**
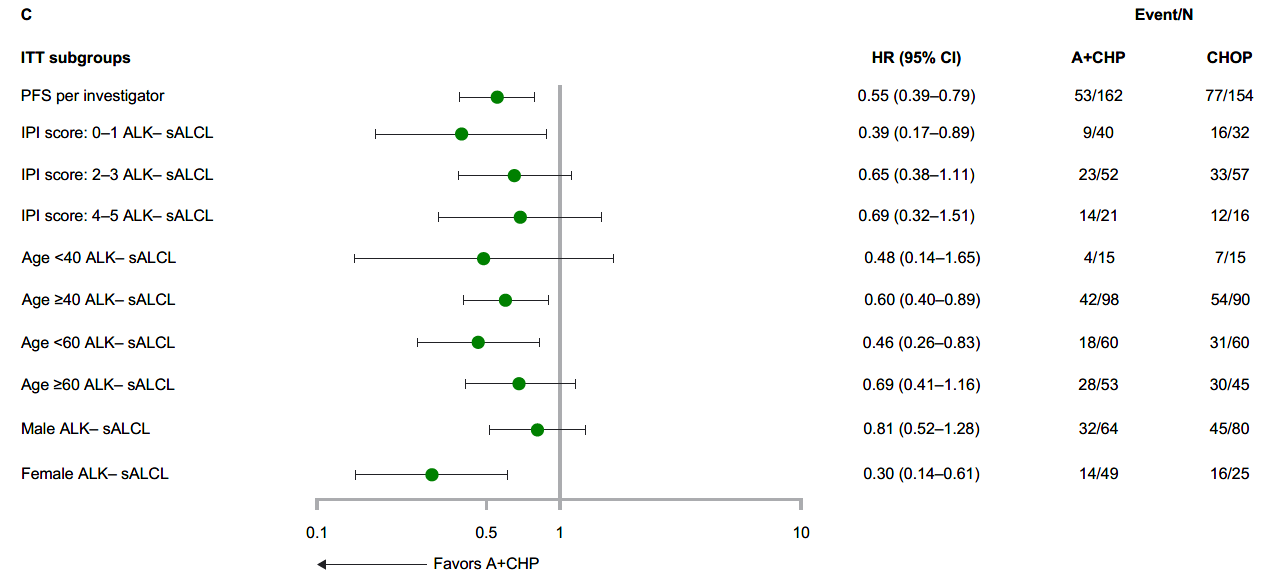
**

^a^HR calculated using stratified cox regression model with ALK status (ALK+/ALK) and IPI score (0–1/2–3/4–5 at randomization as stratification factors. *A+CHP* brentuximab vedotin plus cyclophosphamide, doxorubicin, and prednisone, *ALK* anaplastic lymphoma kinase, *CHOP* cyclophosphamide, doxorubicin, vincristine, and prednisone, *CI* confidence interval, *ECOG* Eastern Cooperative Oncology Group, *HR* hazard ratio, *IPI* International Prognostic Index, *ITT* intent to treat, *NE* not estimable, *sALCL* systemic anaplastic large cell lymphoma.

**Supplementary Figure 3.** Progression-free survival per investigator assessment in patients with sALCL according to ALK status and IPI score* (intent-to-treat population, *N =* 316).


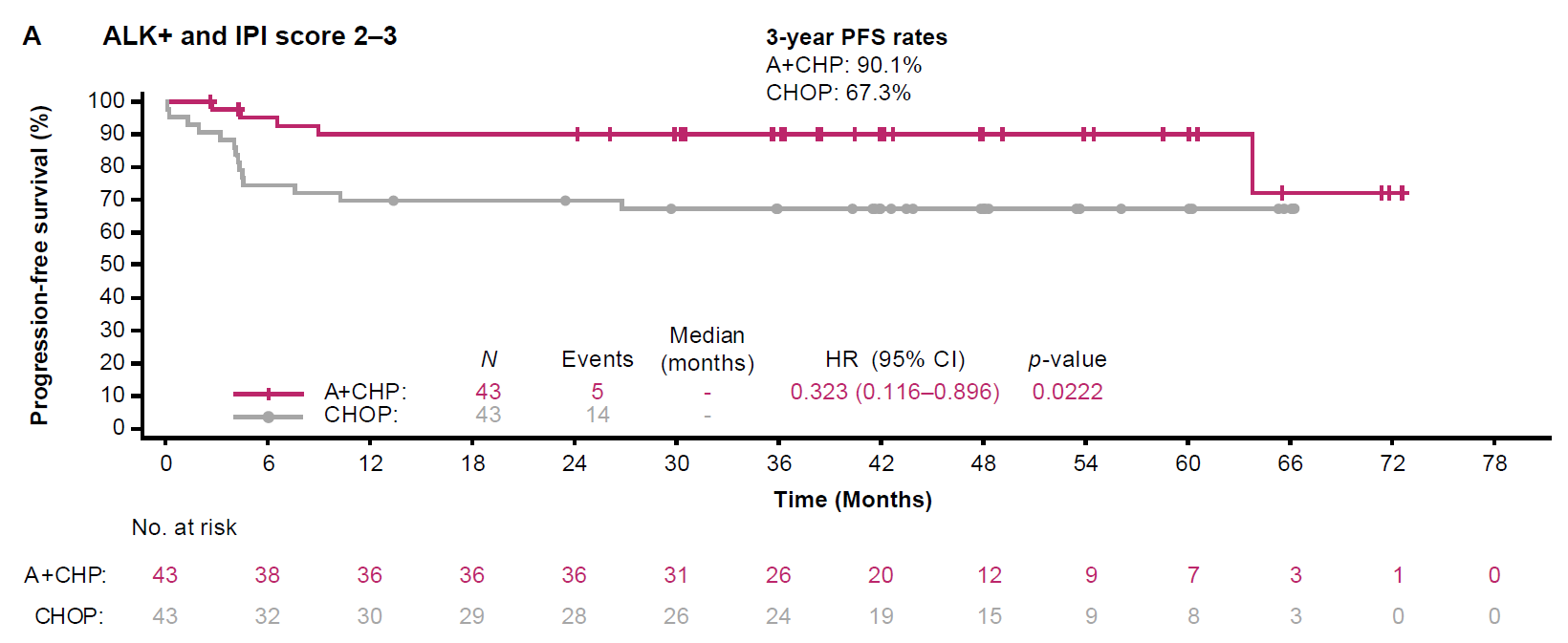


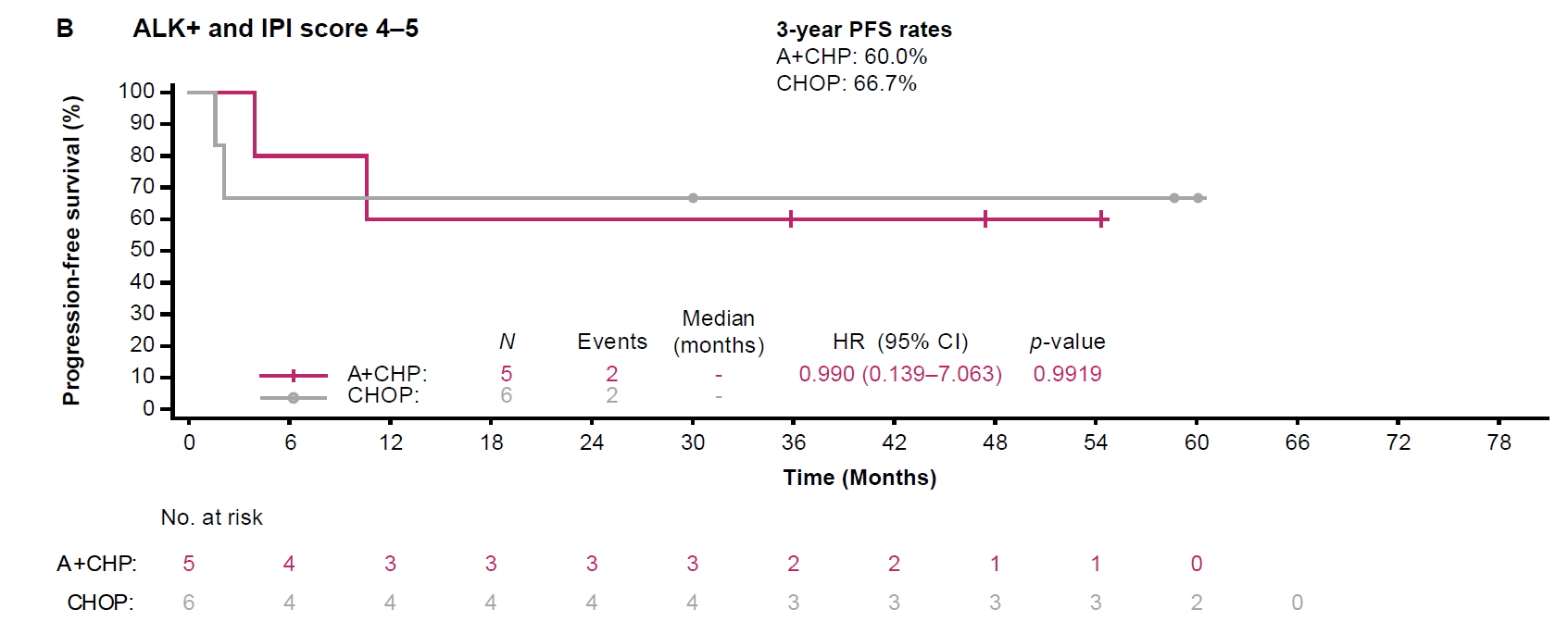


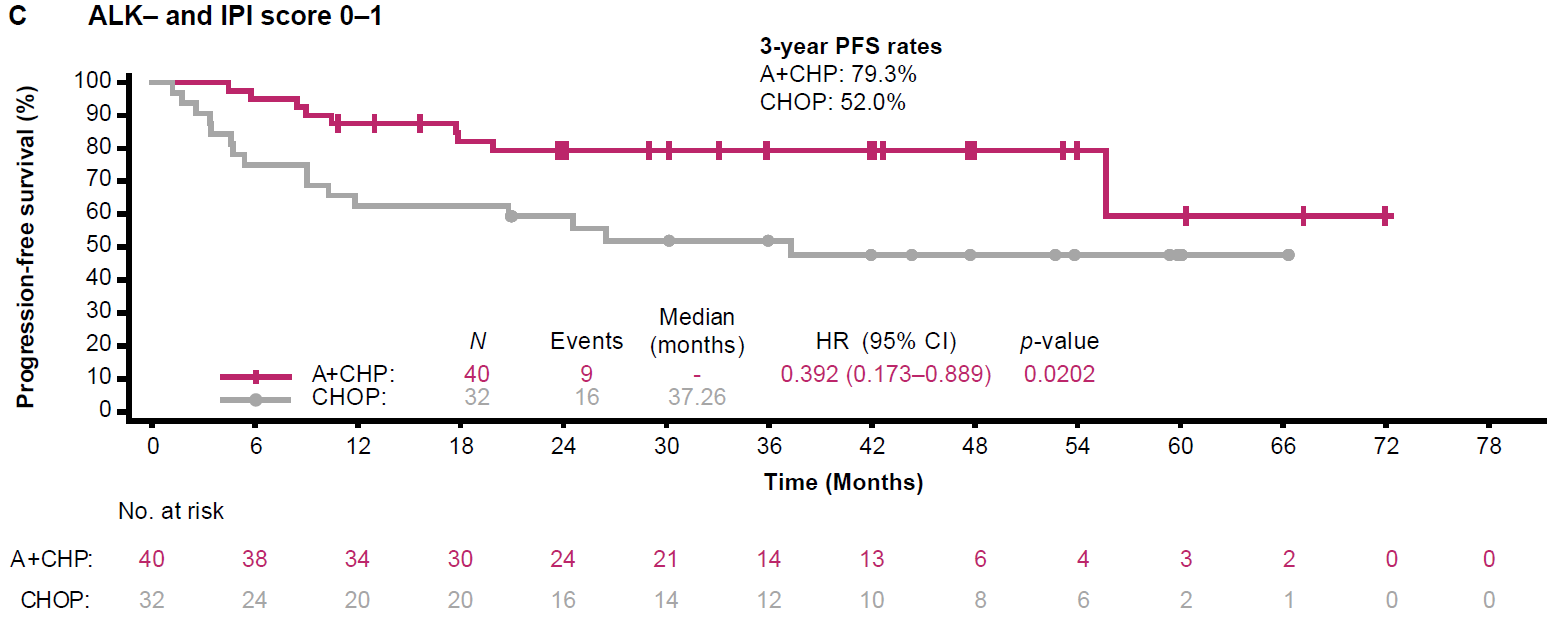


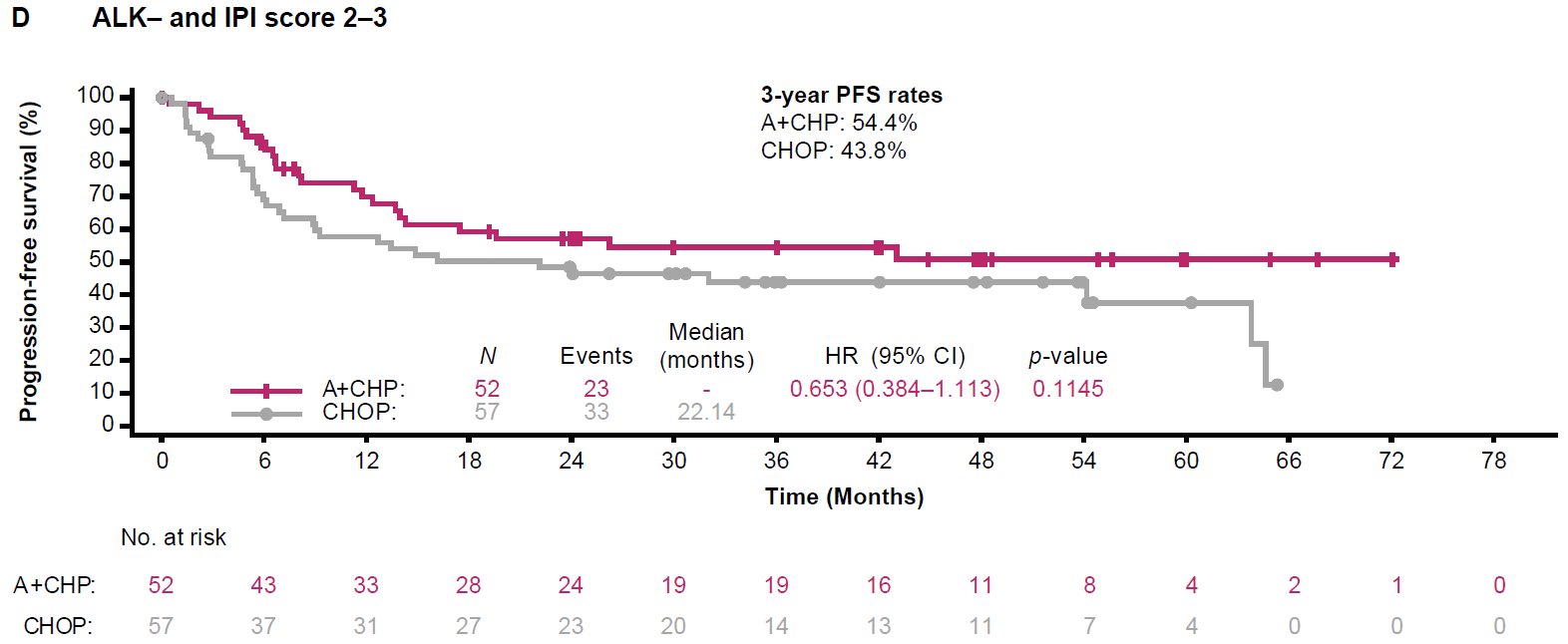


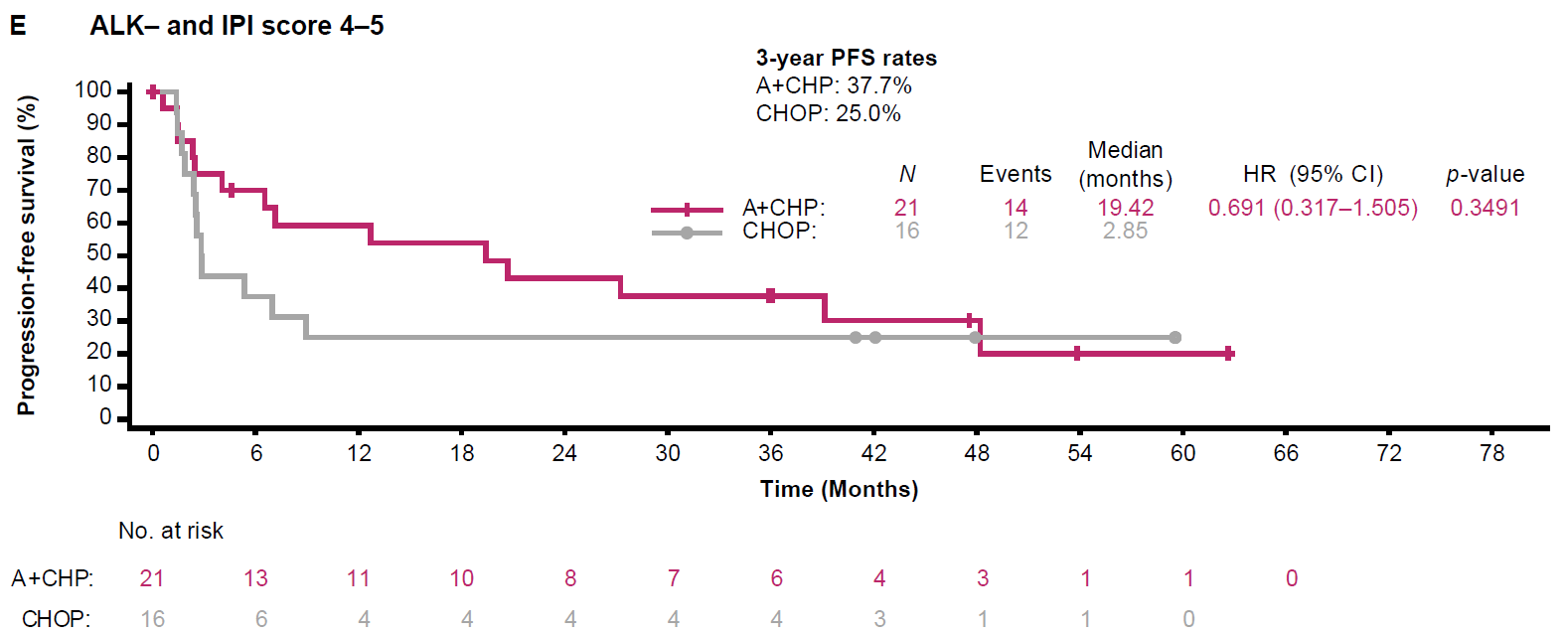


*The ALK+ IPI 0–1 score subgroup contained only 1 patient; therefore, a Kaplan–Meier curve was not generated for this group. *A+CHP* brentuximab vedotin plus cyclophosphamide, doxorubicin, and prednisone, *ALK* anaplastic lymphoma kinase, *CHOP* cyclophosphamide, doxorubicin, vincristine, and prednisone, *CI* confidence interval, *HR* hazard ratio, *IPI* International Prognostic Index, *sALCL* systemic anaplastic large cell lymphoma.

**Supplementary Figure 4.** Overall survival in patients with (A) sALCL, and in the (B) ALK+ and (C) ALK– sALCL subgroups (intent-to-treat population, *N =* 316).


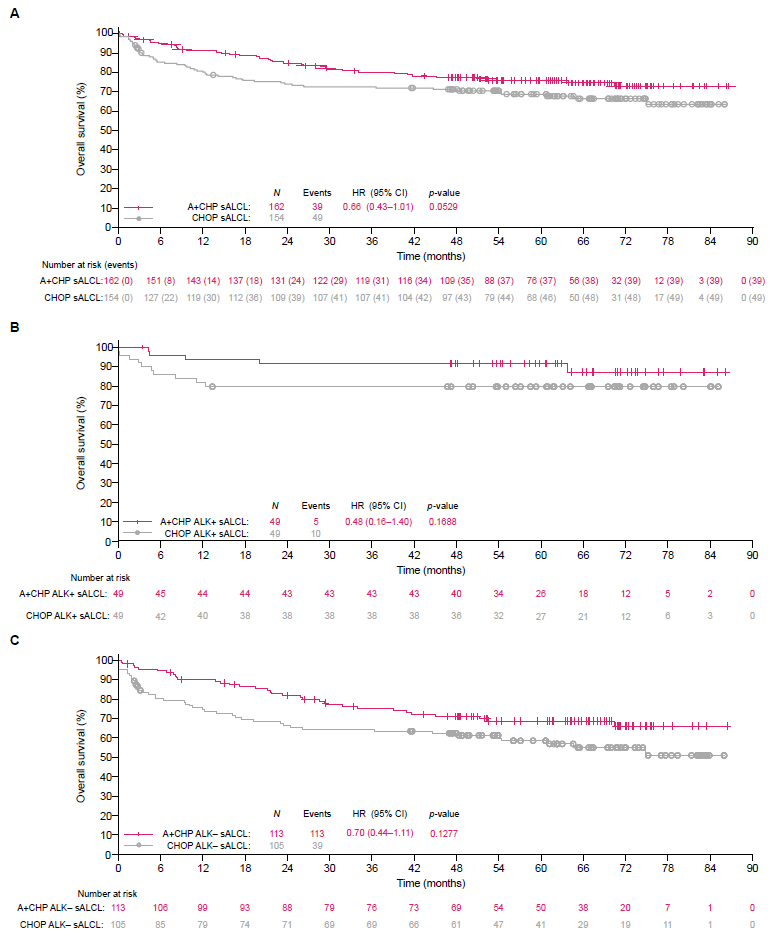
*A+CHP* brentuximab vedotin plus cyclophosphamide, doxorubicin, and prednisone, *CHOP* cyclophosphamide, doxorubicin, vincristine, and prednisone, *CI* confidence interval, *HR* hazard ratio, *sALCL* systemic anaplastic large cell lymphoma.

**Supplementary Figure 5.** Forest plot of hazard ratios for overall survival according to baseline characteristics in (A) patients with sALCL and (B) ALK+ and (C) ALK– sALCL subgroups (intent-to-treat population, *N =* 316).

**
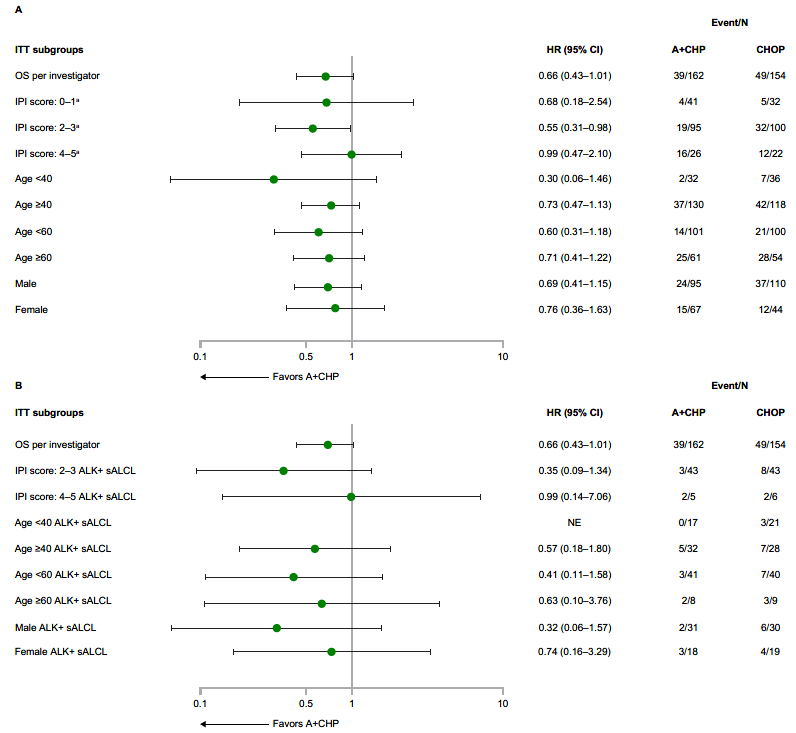
**

**
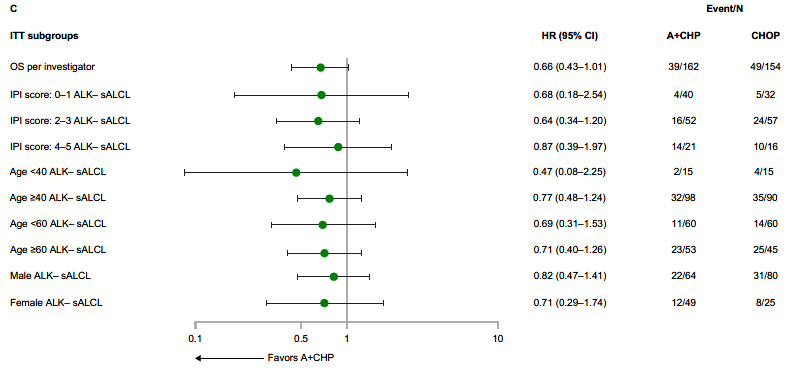
**

^a^HR calculated using stratified cox regression model with ALK status (ALK+/ALK) and IPI score (0–1/2–3/4–5) at randomization as stratification factors. *A+CHP* brentuximab vedotin plus cyclophosphamide, doxorubicin, and prednisone, *ALK* anaplastic lymphoma kinase, *CHOP* cyclophosphamide, doxorubicin, vincristine, and prednisone, *CI* confidence interval, *ECOG* Eastern Cooperative Oncology Group, *HR* hazard ratio, *IPI* International Prognostic Index, *ITT* intent to treat, *NE* not estimable, *PFS* progression-free survival, *sALCL* systemic anaplastic large cell lymphoma.

**Supplementary Figure 6.** Time to subsequent therapy in patients with sALCL (intent-to-treat population, *N =* 316).


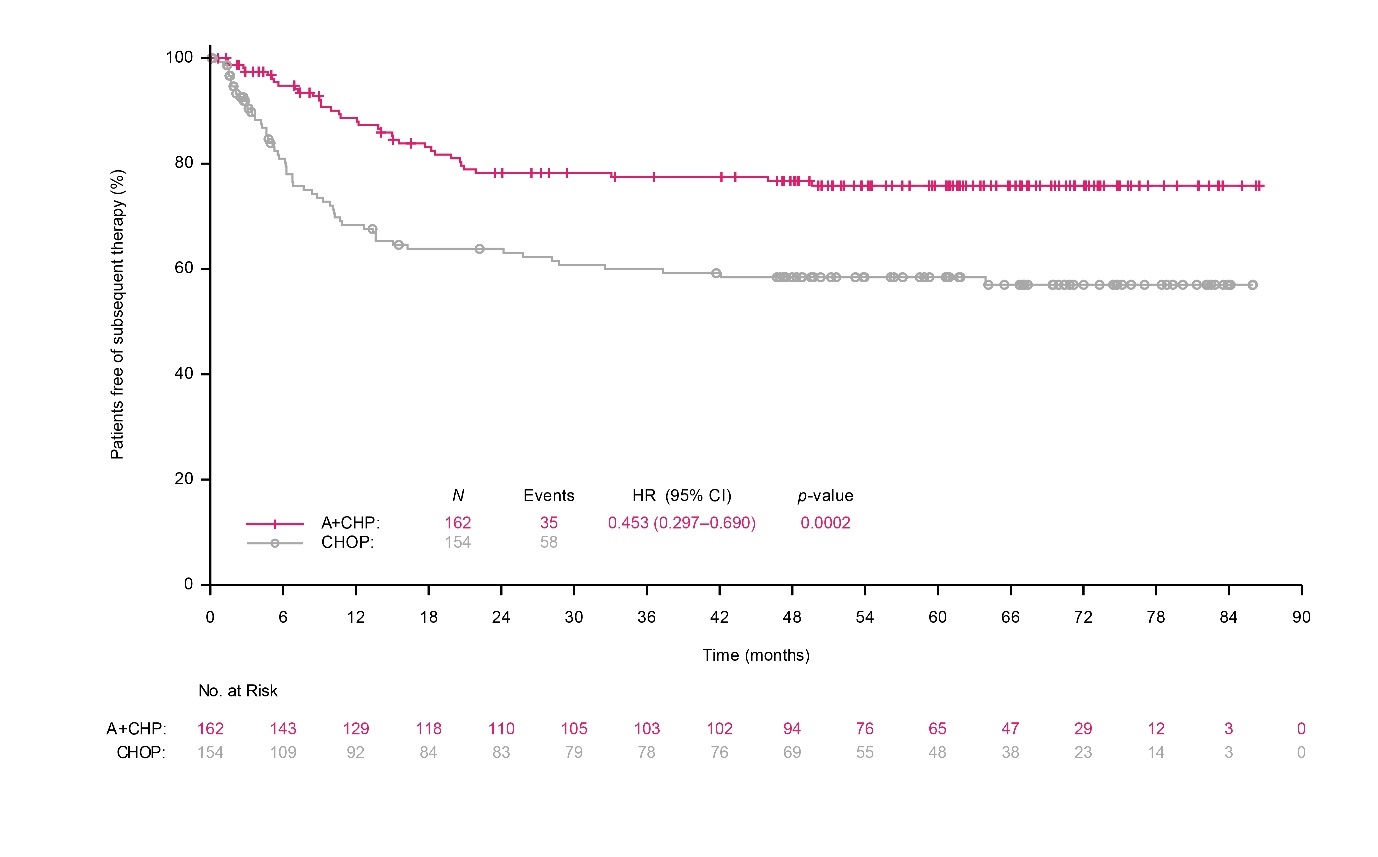


*A+CHP* brentuximab vedotin plus cyclophosphamide, doxorubicin, and prednisone, *CHOP* cyclophosphamide, doxorubicin, vincristine, and prednisone, *CI* confidence interval, *HR* hazard ratio, *sALCL* systemic anaplastic large cell lymphoma.

SUPPLEMENTARY REFERENCES

1. Swerdlow SH, Campo E, Harris NL, Jaffe ES, Pileri SA, Stein H, et al., editors. WHO Classification of Tumours of Haematopoietic and Lymphoid Tissues. Geneva, Switzerland: WHO Press; 2008.
2. Horwitz S, O'Connor OA, Pro B, Trümper L, Iyer S, Advani R, et al. The ECHELON-2 Trial: 5-year results of a randomized, phase III study of brentuximab vedotin with chemotherapy for CD30-positive peripheral T-cell lymphoma. Ann Oncol 2022;33:288–298.
